# Static and Dynamic: Evolving Biomaterial Mechanical Properties to Control Cellular Mechanotransduction

**DOI:** 10.1002/advs.202204594

**Published:** 2023-01-19

**Authors:** Wenyan Xie, Xi Wei, Heemin Kang, Hong Jiang, Zhiqin Chu, Yuan Lin, Yong Hou, Qiang Wei

**Affiliations:** ^1^ Department of Biotherapy State Key Laboratory of Biotherapy and Cancer Center West China Hospital Sichuan University Chengdu Sichuan 610065 China; ^2^ Department of Mechanical Engineering The University of Hong Kong Hong Kong China; ^3^ Department of Materials Science and Engineering Korea University Seoul 02841 South Korea; ^4^ Department of Electrical and Electronic Engineering (Joint Appointment with School of Biomedical Sciences) The University of Hong Kong Hong Kong China; ^5^ Department of Electrical and Electronic Engineering The University of Hong Kong Hong Kong China; ^6^ Institut für Chemie und Biochemie Freie Universität Berlin Takustrasse 3 14195 Berlin Germany; ^7^ College of Polymer Science and Engineering State Key Laboratory of Polymer Materials and Engineering Sichuan University Chengdu 610065 China

**Keywords:** cellular force, ECM dynamics, engineering biomaterials, matrix mechanics, mechanotransduction

## Abstract

The extracellular matrix (ECM) is a highly dynamic system that constantly offers physical, biological, and chemical signals to embraced cells. Increasing evidence suggests that mechanical signals derived from the dynamic cellular microenvironment are essential controllers of cell behaviors. Conventional cell culture biomaterials, with static mechanical properties such as chemistry, topography, and stiffness, have offered a fundamental understanding of various vital biochemical and biophysical processes, such as cell adhesion, spreading, migration, growth, and differentiation. At present, novel biomaterials that can spatiotemporally impart biophysical cues to manipulate cell fate are emerging. The dynamic properties and adaptive traits of new materials endow them with the ability to adapt to cell requirements and enhance cell functions. In this review, an introductory overview of the key players essential to mechanobiology is provided. A biophysical perspective on the state‐of‐the‐art manipulation techniques and novel materials in designing static and dynamic ECM‐mimicking biomaterials is taken. In particular, different static and dynamic mechanical cues in regulating cellular mechanosensing and functions are compared. This review to benefit the development of engineering biomechanical systems regulating cell functions is expected.

## Graphical abstract

The heart of cell–materials interactions is to capture and understand the force crosstalk between cells and materials. However, these force dynamics have not been systemically discussed. Here a biophysical perspective on the state‐of‐the‐art manipulation techniques and novel materials in designing static and dynamic extracellular matrix (ECM)‐mimicking biomaterials is taken. The active force‐biosignaling feedback loops in the dynamic cell–matrix interactions are emphasized.

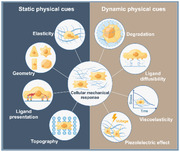

## 1 Introduction

The extracellular matrix (ECM), a complex, dynamic, and crosslinked meshwork with tethered biomolecules, is fundamental to the form and function of tissues and organs. It offers crucial physical support for the cells and generates essential biochemical and biomechanical signals required for tissue development. The ECM is generated through dynamic, reciprocal, biochemical, and biophysical communication between the various cells (e.g., fibroblasts, adipocytes, and stem cells). These interactions between cells and ECM impact various physiological and pathological processes, including homeostasis, aging, wound healing, and various diseases (e.g., cancer, fibrosis, cardiovascular diseases, and pulmonary diseases).^[^ ^1^ ^]^ Intervention in many diseases via manipulating cell‐ECM interactions to control cell behaviors holds great promise for the future.

The cell–ECM interactions are highly dynamic and force‐dependent.^[^ ^2^ ^]^ Specifically, the ECM is constantly being remodeled by cell‐generated forces or externally applied forces, making it a highly dynamic structure. Together with considerable heterogeneity in composition, ECM endows each organ with specific biochemical and mechanical properties, for instance, tensile and compressive strength, topology, and elasticity. Cells experience extrinsic mechanical cues such as shear, tensile and compressive forces and define the cellular behaviors to maintain tissue‐level structural integrity and functionality.^[^ ^3^ ^]^ Cells sense their surroundings through membrane receptors, such as integrins and cadherins.^[^ ^4^ ^]^ When a mechanical load is applied to the adhesion receptors, force‐induced functionalities are activated, for instance, switches in protein conformation or changes in enzyme‐catalyzed reactions (e.g., talin unfolding, FAK activities) that in turn initiates biochemical cues (namely mechanosignaling).^[^ ^5^ ^]^ These biomechanically initiated biochemical cues elicit subsequent cellular responses (e.g., polarity, migration, differentiation, and survival) to adapt to physiological stimuli.^[^ ^6^ ^]^ Therefore, exploring mechanical crosstalk and signaling transduction between cells and ECM mimetics is one of the predominant strategies for studying cell–ECM interactions.

In recent years, ECM‐mimicking biomaterials with various biophysical or biochemical properties have been developed. The mechanical properties of these materials, such as stiffness, viscosity, degradability, and diffusibility, can be precisely controlled, allowing the study of cellular responses against each mechanical property and even combined mechanical cues.^[^ ^7^, ^8^ ^]^ More importantly, novel biomaterials with dynamic properties have been developed with advanced technologies, providing a more realistic microenvironment platform to study cellular mechanoresponse.^[^ ^9^, ^10^, ^11^ ^]^ In this review, we introduce the components of ECM and discuss how cells sense and respond to the individual mechanical properties of the ECM. We overview the state‐of‐the‐art manipulation techniques and novel materials in designing static and dynamic ECM‐mimicking biomaterials. In particular, we summarize different static and dynamic mechanical cues in regulating cellular mechanosensing and functions.

## 2 Cell–Material Interactions

### 2.1 Extracellular Matrix (ECM)

**Figure 1:**
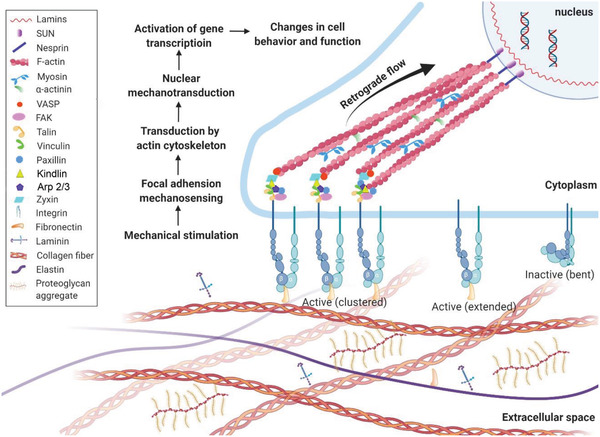
Cellular mechanosensing. This process includes mechanical stimulation, force transmission through actin filament—proteins chains, mechanical signaling conversion, and activation of transcriptional factors and transcripts, and finally, guides the cell functions and phenotypes.

The native ECM supplies surrounding cells' structural and mechanical support and mediates diverse cell behaviors, including adhesion, migration, proliferation, self‐renewal, quiescence, differentiation, etc., through biochemical signals and physical cues.^[^ ^12^, ^13^, ^14^ ^]^ In mammals, the ECM is composed of an interlocking mesh of fibrous proteins and polysaccharides. The fibrous proteins, e.g., elastin, collagen, fibronectin, laminin, etc., confer the ECM with tensile strength, elasticity, and adhesive ligands.^[^ ^15^, ^16^ ^]^ The hydrated polysaccharide gel containing glycosaminoglycans (GAGs) and proteoglycans (PGs) confer the ECM with viscoelasticity (**Figure** **1**).^[^ ^17^, ^18^ ^]^

Elastic fibers are the most biochemically and physically stable constituent in the ECM.^[^ ^19^ ^]^ They consist of an elastin core embraced by a sheath of microfibrils, including glycoproteins fibrillin and fibulin.^[^ ^15^ ^]^ These elastic fibers provide connective tissues, including blood vessels, lungs, and skin with essential features of extensibility and resilience, facilitating long‐range deformability and passive recoil without energy input and thereby responsible for mechanical memory (ability to recoil back to their steady state).^[^ ^20^ ^]^

In mammals, collagen is the most abundant protein and accounts for approximately 30% of total protein mass.^[^ ^21^ ^]^ This protein family contains 28 members numbered with Roman numerals.^[^ ^22^ ^]^ As a crucial structural element in ECM of most connective tissues, including ligaments, tendons, bone, and cartilage. Collagen offers toughness and tensile strength, providing mechanical cues and topographic cues (e.g., porosity and hierarchical structures) to regulate cell functions.^[^ ^22^ ^]^ For instance, collagen in the tendons shapes thick fibers (200 nm). They arrange along the tendon to facilitate force transfer and support tendon strength.^[^ ^23^ ^]^ On the contrary, thin collagen fibers (30 nm) in the cornea are woven together to generate strength and optical transparency.^[^ ^24^ ^]^ In addition, the remodeling of collagen fibers is a continuous process during the whole life. It keeps providing successive dynamic stimulations to the embedded cells.^[^ ^25^ ^]^ The turnover of collagens is a dynamic process, and collagen accumulation depends on its synthesis and degradation. The remodeling and equilibrium between the synthesis and degradation of ECM components contribute to its homeostasis. Therefore, the structural and functional integrity of ECM relies not only on fiber density, orientation, crosslinking, and interactions between ECM components but also on the remolding and turnover of ECM components, especially under stressful circumstances.^[^ ^19^ ^]^

GAGs are linear polysaccharides comprised of repeating disaccharide units. PGs are core proteins covalently bound with GAG side chains. PGs form a gelatinous and hydrated substance interspersed among the fibrous proteins in connective tissues. GAGs, especially heparin sulfates, can bind various proteins (such as enzymes, growth factors, and cell adhesion proteins) and result in the immobilization of proteins in the ECM, regulating physiological activities.^[^ ^26^ ^]^

Adhesive ECM glycoproteins, including fibronectin, laminins, vitronectin, and others, interact with other ECM proteins to create a mighty matrix network. They are involved in the interaction between cells and ECM by acting as ligands for cell surface receptors such as integrins and in turn, mediate related cell behaviors and functions.^[^ ^27^ ^]^

Over the last decade, the influence of physical properties of ECM on cell behaviors has been extensively studied, such as stiffness (the resistibility of an object to deformation), elasticity (a property of a material that causes it to be restored to its origiinal state after deformation),^[^ ^28^, ^29^ ^]^ viscoelasticity (the resistance of a fluid to a deformation at a given rate),^[^ ^30^ ^]^ roughness,^[^ ^31^ ^]^ scaffold dimensionality (2D or 3D),^[^ ^32^ ^]^ thickness^[^ ^33^ ^]^ and so on. These parameters profoundly affect cell adhesion, migration, proliferation, and differentiation. The developing knowledge in cell‐ECM interactions will guide the adjusting of ECM properties to modulate cell behaviors and functions.

### 2.2 Cell Mechanosensing

Cells can sense and respond to the biophysical cues of the microenvironment in a process called mechanotransduction, which integrates and converts biophysical cues in microniche to intracellular biochemical signals.^[^ ^13^, ^19^ ^]^ This rapid conversion of physical to biochemical response enables cells to rapidly adapt to the physical environment and thereby maintain their mechanohomeostasis (Figure 1). This process involves two main steps: force transmission and mechanotransduction.^[^ ^34^ ^]^ Force transmission refers to the transmission of mechanical forces through cellular components, including actin stress fibers, microtubules, and other related molecules.^[^ ^35^ ^]^ Specifically, the physical signals are detected by cells mainly through integrins or cadherins, two principal transmembrane adhesion receptors that direct the mechanical link between the ECM and the cell cytoskeleton,^[^ ^6^ ^]^ and are balanced by myosin‐induced intracellular force.^[^ ^34^ ^]^ The force can be transmitted along with the receptors and cytoskeleton to the nucleus at a speed of up to 30 µm s^−1^, which is 2.5 times faster than the signal transduction mediated by passive diffusion of signaling molecules.^[^ ^1^, ^13^ ^]^ Mechanotransduction refers to the actual process of conversion of physical signals into biochemical signals, which typically involves the conformation changes of the mechanosensitive proteins (e.g., integrin, FAK, etc.), and the activation of downstream biochemical signaling pathways and genetic transcription.^[^ ^6^ ^]^ However, how these mechanosensitive proteins sense and trigger the downstream signaling events opens up many intriguing questions.

Cells must sense and adjust ECM mechanics to maintain mechanical homeostasis and keep tissue structurally integrity and functionality.^[^ ^19^ ^]^ The force transmission across adhesive proteins on cellular membrane establishes mechanical reciprocity between cell microenvironment and cellular tension. During force loading processes, cell surface adhesion proteins are activated to initiate biochemical signals that regulate rapid cellular mechanical responses and long‐term changes in gene expression.^[^ ^12^, ^36^, ^37^ ^]^ In this review, we mainly discuss how engineering materials regulate cellular mechanics and function through integrin‐mediated cell adhesions. For mechanotransduction mediated by other adhesion receptors (e.g., cadherin), please refer to the relevant references.^[^ ^38^, ^39^ ^]^

### 2.3 Integrin‐Mediated Cell Adhesion and Mechanosensing

Integrins are transmembrane adhesion proteins that recognize and bind to specific ECM proteins/peptides and cellular counter receptors.^[^ ^40^ ^]^ They connect ECM to the intracellular actin cytoskeleton and establish mechanical links between extracellular and intracellular compartments. They are heterodimeric transmembrane proteins consisting of *α* subunit and *β* subunit. There are 18 *α*‐subunits and 8 *β*‐subunits forming at least 24 unique combinations in mammals, each recognizing a specific set of ECM ligands.^[^ ^6^, ^41^ ^]^ For example, *α*5*β*1 and *α*v*β*3 integrins recognize the RGD motif in various ECM proteins such as fibronectin and vitronectin; collagen can bind integrin *α*2*β*1, *α*10*β*1, and *α*11*β*1.^[^ ^42^ ^]^

**Figure 2:**
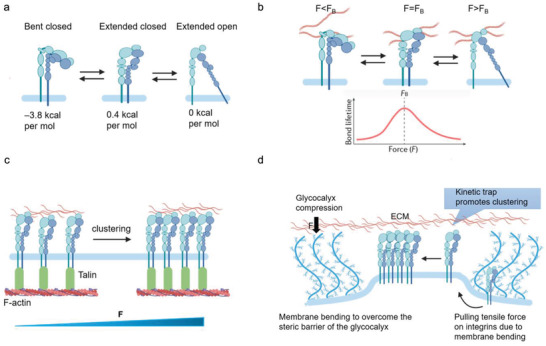
Integrin activation and integrin–ligand force crosstalk. a) The integrin conformation changes integrin and their thermodynamics during activation. b) Integrin–ligand interaction follows a catch–slip bond transition. When the force *F* is applied to the ligand‐bound integrin is lower than the optimal bond force (*F*_B_), the bond lifetime rises with force. When *F* surpasses *F*_B_, the bond lifetime declines with force. c) External force can trigger clustering. d) Integrin clustering regulated by glycocalyx.

Immature integrins are transported to the plasma membrane as an inactive heterodimer of bent conformation with a closed headpiece (**Figure** **2**a). The ECM binding allows integrin to unbend and the headpiece open, resulting in activation of integrin and an increase in ligand affinity.^[^ ^43^ ^]^ These outside‐in signals recruit F‐actin binding adaptor proteins, including talin, paxillin, and vinculin, and facilitate the connection between integrins and cytoskeleton.^[^ ^44^ ^]^ Reciprocally, actomyosin pulls on integrins, further contributing to the force generation. Inside‐out signals can also activate integrins through the displacement of intracellular integrin inhibitors, which allow intracellular adapters (such as talin) to bind to integrin *β*‐subunits, therefore, regulating integrin affinity for ECM ligands. This model is called “integrin inside‐out activation.” During this process, talin and kindlin play vital roles in integrin activation. The talin rod domain contains several binding sites for actin and vinculin, some binding sites only can be exposed when applied forces over a certain threshold.^[^ ^45^ ^]^ However, it seems that the binding of talin solo is insufficient for complete integrin activation.^[^ ^46^ ^]^ Recent evidence shows that kindlin is required for full activation of integrin. Unlike talin, kindlin 2 and 3 bind to the integrin tails at the NxxY motif and recruit the integrin‐linked kinase (ILK)‐PINCH‐parvin complex, paxillin, and the Arp 2/3 complex to integrins.^[^ ^47^, ^48^ ^]^ The resulted complexes may enable the activation of FAK, Rac 1, and Arp 2/3 complex through the paxillin‐*β*‐Pix axis and therefore promote cell spreading.^[^ ^48^, ^49^ ^]^ Kindlin 2 can interact with the ILK and migfilin (FBL) which are directly linked to the actin cytoskeleton.^[^ ^50^, ^51^ ^]^ Interestingly, no intramolecular tension between the kindlin and actin has been found yet.^[^ ^44^ ^]^

As previously discussed, the integrin activation is highly talin‐ and kindlin dependent. The classical “integrin inside‐out activation” model highlights talin's unique and striking role in inducing integrin conformational changes. However, it cannot give a clear thermodynamics landscape of the conformation changes in integrin activation, that is—whether and how the binding of talin and kindlin to the integrin tail can overcome the energy barriers between the bent‐closed state and extended‐open state (Figure 2a). Recent evidence indicates that the bent‐closed state of *α*5*β*1 contains the lowest free energy of ‐3.8 kcal mol^−1^ compared to the extended‐closed state (0.4 kcal mol^−1^) and the extended‐open states (0 kcal mol^−1^). The significant gaps in free energy make the *α*5*β*1 favor greater the bent‐closed state. Whereas the ligand‐binding affinity of the extended‐open conformation of *α*5*β*1 is 5000‐fold higher than that of bent‐closed or extended‐closed state.^[^ ^52^ ^]^ Thus, the primary energy barrier for *α*5*β*1 integrin activation is enough to overcome the bent‐closed state. Once achieved, the extended‐closed state will shift to the high‐affinity extended‐open conformation due to its tiny free energy difference.

Recently, a new model, named “thermodynamic equilibrium,” indicates that talin and kindlin may transduce small mechanical force to the integrin‐ligand complex to maintain the rare extended‐open integrin state instead of interfering with the transmembrane association between integrin *α*‐ and *β* subunits.^[^ ^53^ ^]^ The force driven via talin and kindlin may speed up conformational changes and shift the thermodynamic equilibrium of integrin conformations to the extended‐open state.^[^ ^44^ ^]^ According to Li and Springer,^[^ ^53^ ^]^ the increase of concentration of the active states of adaptors (e.g., talin) that link integrins to the actin cytoskeleton may also enable integrins to maintain their rare extended open state, besides the force.^[^ ^54^ ^]^ However, it is hard to believe that cytosolic talin could reach such a high concentration to stabilize the integrin extended‐open state since talin is recruited in FAs directly from the cytoplasm instead of enrichment on the plasma membrane^[^ ^54^ ^]^ and the lower affinity to the integrin tails.^[^ ^55^ ^]^

Ligand‐binding interactions exhibit a certain behavior termed “catch‐slip bond.” For a catch–slip bond, the applied force first strengthens the bond (catch regime), and once the force exceeds a certain threshold, it begins to weaken the bond (slip regime), resulting in a decay of bond lifetime with applied force (Figure 2b). This is due to the different bond configurations in different force loading states.^[^ ^44^ ^]^ In addition, their interaction dynamics vary within different integrins. Their diverse binding affinities to talin or kindlin result in different energy profiles when binding with identical ligands.^[^ ^56^, ^57^ ^]^ For instance, *α*4*β*1 is easier to be activated than *α*5*β*1. However, its high‐affinity binding to cell adhesion molecules and fibronectin is 100–1000 folds weaker than the binding between *α*5*β*1 and fibronectin. And its difference in ligand binding affinity between the extended‐open state and bent‐closed state (600–800 folds) is more compressed than that of *α*5*β*1 (5000 folds).^[^ ^56^ ^]^ The activation of *α*L*β*2 integrin also exhibits a more considerable conformational change that correlates with force coupling, as a 1–6 pN range of a force is indicated to be associated with binding to the ligand and the cytoskeleton in T cells.^[^ ^58^, ^59^ ^]^

When integrins bind with extracellular ligands and transform from a low‐ to a high‐affinity conformation, either by an outside‐in (integrin activation via binding to the ECM ligands at the integrin extracellular domain) or inside‐out (the cytoplasmic tail of integrin is activated by cytoplasmic proteins, including talin or kindlin) mechanisms, they start to form nanosized clusters (crosslinked to each other and actin through adaptor proteins) within the membranes and further assemble into larger integrin clusters to increase the local adhesion strength.^[^ ^60^ ^]^ The integrin clustering is also mechanosensitive (Figure 2c). A given integrin will be subjected to a given elastic strain during the clustering mechanosensing process when an external force is applied. Thus, it is predictable that the integration of an additional integrin into the cluster will be energetically favorable due to the decrease in the tension of individual integrin molecules.^[^ ^61^ ^]^ More importantly, the mechanical properties of a substrate could be a significant factor in regulating the lateral diffusion ability of integrins to form clustering, which might be caused by substrate‐based integrin restriction.^[^ ^62^ ^]^ Integrin clustering allows integrins temporarily detach from the ligand by intensive forces to rapidly rebind to the ligand, which considerably prolongs the lifetime of the adhesions.^[^ ^6^ ^]^ This process is associated with the kinetics of the integrin‐ligand rebinding and the conformation switch between extended‐open state and thermodynamically favored bent‐closed state. However, neither timescale of these two states has been demonstrated yet.^[^ ^44^ ^]^ Interestingly, the glycocalyx, a dense layer of complex polysaccharide complex on the cell surface with more than 20 nm thickness,^[^ ^63^ ^]^ could promote integrin clustering.^[^ ^64^ ^]^ To overcome the physical glycocalyx barrier and get access to the ligands, cells destabilize the cortical actin structures to mobilize glycocalyx receptors and compress the glycocalyx by applying protrusive force to deform membrane with protrusion structures, including filopodia, lamellipodia, and podosomes.^[^ ^65^, ^66^ ^]^ At the same time, adhesome is preactivated by enriched adaptor proteins, which makes integrins easily captured and mechanically engaged.^[^ ^64^ ^]^ The deformed membrane generates a mechanical resistance that results in a pulling force on the integrin and a compressive force on surrounding glycoproteins. Specifically, the force applied to the integrin will lead to changes in integrin conformation or ligand‐integrin binding kinetics. The compression of glycocalyx around these pre‐formed integrin–ligand complexes results in a much closer distance between diffusing integrins and substrate, thereby facilitating these integrins higher accessibility to the binding sites.^[^ ^64^ ^]^ These properties together promote integrin‐based cell adhesion strengthening and signaling (Figure 2d).

The integrins act as the transmembrane “bridge” that allows the force‐talk between the cell and the matrix bidirectionally. Once an integrin binds to its ligand, the force is applied. It can be transmitted between the ECM and the actomyosin cytoskeleton through a molecular clutch, “ECM‐integrins‐adaptor proteins‐actin”. For example, any force applied to ECM pulls on ligand‐binding integrins. The integrin cytoplasmic tails bind to actin‐binding proteins and transmit applied forces to the actomyosin cytoskeleton. Vice versa, the forces generated by myosin contraction or actin polymerization apply to actin and transfer to ECM via actin‐binding proteins and integrins.^[^ ^67^, ^68^ ^]^

### 2.4 Molecular Clutch

**Figure 3:**
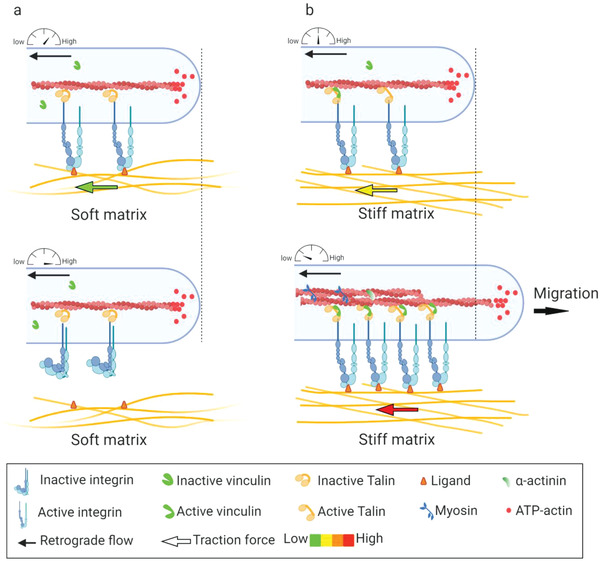
Force transmission through the molecular clutch. a) On soft substrates, the force loading rate is slower than the lifetime of integrin–ECM bond, leading to a bond dissociation before talin unfolding or vinculin binding. b) On stiff substrates, the force is loaded faster than the integrin–ECM bond lifetime, resulting in talin unfolding, vinculin binding, and actin‐based reinforcement.

Generally, the mechanical linkage between the ECM‐binding integrins and the force‐generating actomyosin cytoskeleton is proposed as the molecular clutch. Despite the molecular complexity of cell–matrix adhesions, the elementary constituents of a molecular clutch for force transmission can be sorted as 1) actin filaments; 2) myosin motors exerting force on actin filaments; 3) adaptor proteins; 4) integrins, and 5) ECM ligands (**Figure** **3**). Integrin‐ and F‐actin‐binding proteins (e.g., talin and vinculin) involved in the clutch that connects integrins to actomyosin cytoskeleton. Talin plays a critical role among the proteins involved in the molecular clutch associated proteins. As previously discussed, talin is a mechanosensitive protein that connects actin to the ECM via integrins.^[^ ^69^ ^]^ Its conformation changes when the force loading exceeds a certain threshold, allowing vinculin binding. The additional recruitment of vinculin reinforces the clutch and thereby strengthens the labile talin‐mediated linkage between integrins and actin. Other molecules such as kindlin, filamin, and *α*‐actinin may also associate with the molecular clutch.^[^ ^70^ ^]^

The molecular clutch is a particularly dynamic complex that serves as a mechanosensitive linkage between matrix and cells.^[^ ^6^ ^]^ Specifically, the cellular mechanosensitive molecular activities such as bond rupture or protein unfolding have proved to be associated with the force loading rate (a rate at which applied force increases to a clutch). The force loading rate on the clutches governs mechanosensing by increasing reinforcement and adhesion formation in an integrin and talin‐dependent way (e.g., integrin‐ECM binding kinetics, talin unfolding). This theory has been utilized to reveal how cells generate and transmit forces to respond to ECM mechanical cues, for instance, rigidity,^[^ ^71^, ^72^ ^]^ viscosity,^[^ ^73^ ^]^ and ligand presentation.^[^ ^74^ ^]^ On stiff substrates, fast mechanical loading allows engaged clutches to rapidly reach their breaking strength and induce destabilization and disengagement, resulting in decreased force transmission and a higher retrograde flow rate. However, as loading rates increase further, unbinding forces in individual clutch start surpassing the threshold force required for mechanotransduction (reinforcement), resulting in the engagement of additional integrins and improvement of force transmission (Figure 3b). On soft substrates that can be easily deformed, the force builds so slowly that clutches eventually disengage before significant forces can be loaded (Figure 3a).^[^ ^72^, ^75^, ^76^, ^77^ ^]^

### 2.5 Focal Adhesion‐Mediated Force Transmission and Transduction

Focal adhesions (FAs) are large protein complexes assembled at the basal surface of cells. They physically link the ECM to the cytoskeleton. The FA assembly is initiated when enough force is applied to ligand‐binding integrin, either through an outside‐in or inside‐out mechanism. It includes a complex high‐affinity conformation change of integrins. Following such events, integrins are then activated, aggregated into clusters, and reinforce the molecular clutch at the cell–matrix interface.^[^ ^78^ ^]^ The extracellular domain of integrin binds to the matrix ligands, while the cytoplasmic tail domain links to the cytoskeletal actin through numerous recruited proteins, which form the inner core of FAs. The initial integrin clustering occurs underneath the protrusive parts of cell (such as filopodia or lamellipodia), termed “nascent adhesions.”^[^ ^44^ ^]^ They disassemble rapidly if no force is applied. Once the adhesions are connected by bundles of actin filaments (stress fibers), they will grow and elongate into FAs that anchor the cells.^[^ ^79^, ^80^ ^]^

The initial assembly of FAs does not require myosin motor activity.^[^ ^81^ ^]^ It can be mediated through coupling integrins to the actin filaments along with the incorporation of adapter proteins by altering their conformation and biochemical properties.^[^ ^70^ ^]^ For instance, talin molecules unfolding response to the loading force and expose binding sites for vinculin, resulting in additional recruitment of vinculin. Vinculin can bind to actin and reinforce the talin‐mediated linkage between integrins and actins.^[^ ^75^, ^76^ ^]^ Additionally, the paxillin, binding to activated vinculin, exposes extra binding sites for adaptor molecule Crk upon its phosphorylation, which, sequentially, initiates the MAPK signaling cascade and subsequently enhances the stabilization of FA‐cytoskeleton structures.^[^ ^82^, ^83^ ^]^ Other proteins, such as *α*‐catenin and filamin can also alter binding partner affinities under force application, resulting in changes in integrin conformation,^[^ ^84^ ^]^ ion channel activity,^[^ ^85^ ^]^ and enzyme activity.^[^ ^86^ ^]^ Further actomyosin contraction and pull on a stiff ECM (through integrin‐ligand binding) will finally promote the maturation of FAs.^[^ ^87^, ^88^ ^]^

Recently, with the development of super‐resolution microscopy technology, the architecture of mature FAs has been identified by a 3D interferometric photoactivated localization microscopy (iPALM).^[^ ^90^, ^91^ ^]^ The mature FAs consist of several molecular layers. The layer close to the membrane is termed the integrin signaling layer and contains the critical FA signaling proteins FAK and its adapter protein paxillin. The upper layer is called the force transduction layer, a talin‐ and vinculin‐rich area that links the integrin complex to the actomyosin. The third layer within FAs is the actin regulatory layer and actin stress fiber layer consisting of actin and actin‐binding proteins such as *α*‐actin.^[^ ^89^ ^]^ The visualization of the spatial arrangement of FA proteins by iPALM indicates the predetermined location and function of each FA protein.

FAs exhibit mechanosensitive properties and transmit mechanical force between the cytoskeletal contractile machinery and the extracellular matrix, which are prerequisites for cell spreading and migration. The adhesion complexes are highly dynamic. Any physical force can directly or indirectly change the dynamics and interaction of FA proteins to alter the FA composition, morphology, or signaling, resulting in downstream changes in FA‐dependent cellular functions such as FA strengthening and nuclear gene expression.^[^ ^77^ ^]^ The force generated by the myosin‐powered contractility and the continuous actin polymerization drives a constant flow of actin termed “retrograde flow” moving from the cell edge (e.g., lamellipodia) to the cell center (stress fibers). When the machinery is initiated, the force transmitted to the ECM counters myosin contractility, delaying actin retrograde flow, fostering actin protrusion away from the center of the cell, and generating rearward traction forces (the forces that cells exert on their surroundings) by which the cells can moved forward (Figure 1).^[^ ^70^, ^77^ ^]^

FAK plays a central role in regulating FA assembly and disassembly, and thereby acts as the unique controller for the directional cell movement.^[^ ^90^ ^]^ Upon activation, FAK Tyr‐397 (FAKY397) becomes autophosphorylated, which displays a high‐affinity binding site for the Src homology 2 (SH2) domain of Src and related kinases.^[^ ^91^ ^]^ Upon binding to Tyr‐397, Src phosphorylates FAK at several other tyrosine residues and serves as a binding site for other signaling molecules, such as Grb2, p130cas, and phosphatidylinositol 3‐kinase, resulting in the activation of extracellular signal‐regulated kinases (Erks).^[^ ^91^, ^92^ ^]^ Recently, a mathematical model has been established to reveal how the integrin clusters serve as mechanotransduction machinery to convert ECM signaling (e.g., substrate stiffness) into biochemical signaling (phosphorylation of FAKY397).^[^ ^93^ ^]^ The simulation results indicate that the integrin clustering dynamics is highly dependent on integrin type. Different integrins possess varied integrin‐ligand binding affinity, leading to varying lifetimes of integrin clustering. More importantly, a stiff substrate promotes more and longer‐lived integrin clustering, while increased integrin clustering linearly enhances the phosphorylation of FAK. Because this increased density of integrin clustering enables a prolonged reaction time for FAKY397 phosphorylation through increasing the probability of FAK rebinding integrin by talin.^[^ ^92^ ^]^

### 2.6 Nuclear Mechanics

**Figure 4:**
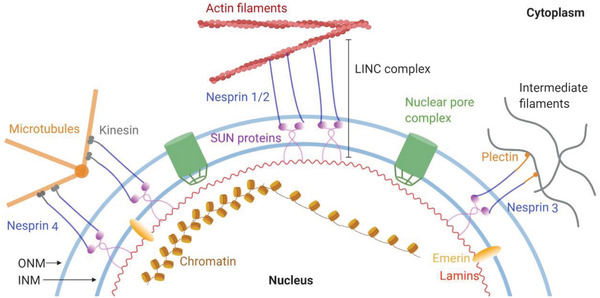
Molecular connectivity from the ECM to the nucleus. The larger isoforms of nesprin 1/2 contain N‐terminal actin‐binding domains, while nesprin 3 and 4 directly bind to cytoskeletal cross‐linkers plectin and kinesin, respectively. At the nuclear envelope, nesprins interact through their C‐terminal KASH domain with SUN proteins. At the inner nuclear membrane, SUN proteins link to lamins and other nuclear envelope proteins. This protein complex formed by the association of SUN proteins and nesprins act as the physical link connecting the nucleus and the cytoskeleton.

The forces applied to the cell membrane activate the membrane signaling events and promote structural rearrangements deeply in the cytoplasm and nucleus. These mechanosensitive cellular components, such as integrin sets, adhesion complex, actomyosin contractile machinery, the linker of nucleoskeleton and cytoskeleton (LINC) complex, and subnuclear molecules, are physically connected (**Figure** **4**).^[^ ^13^, ^94^, ^95^ ^]^ Such a “hard‐wired” mechanism for direct nuclear mechanotransduction allows a much faster physical propagation (5 µs) compared to that of chemical diffusion (approximately 5 s) or translocation‐based signal propagation (5–10 s).^[^ ^96^ ^]^

The LINC complex physically links the nucleoskeleton to the nuclear membranes and the cytoskeleton and provides homeostasis of nuclear anatomical morphology and mechanosensory functions in cells. Its core is a SUN protein located in the inner nuclear membrane (INM) and a nesprin protein found in the outer nuclear membrane (ONM). On the nucleoplasmic side, SUN proteins bind to lamins and INM‐associated proteins such as emerin. On the cytoplasmatic side, the nesprins contact cytoskeleton components, such as actin microfilaments, microtubes, and intermediate filaments (Figure 4). The cells with absent or disrupted nesprins fail to respond to mechanical force.^[^ ^35^ ^]^ Recent evidence indicates that SUN2 serves as a safety valve that affords protection for DNA to resist damage induced by an external force. The overloading force may cause a rapid loss of SUN2 from the nuclear envelope, which decreases the strain‐induced changes to nuclear texture, and thereby desensitizes the lamina and chromatin stability to mechanical stimulations.^[^ ^97^ ^]^

Lamins are the intermediate filament‐like proteins located on the nucleoplasmic surface of the inner nuclear membrane, providing the nucleus the structural integrity. They contribute to nuclear stiffness and stability and play a central role in mechanotransduction. Lamins can be classified into A‐type lamins and B‐type lamins. The most common A‐type lamins are lamins A and C, encoded by the *LMNA* gene and derived by alternative splicing. While B‐type lamins have two subtypes, lamin B1 and B2, which are encoded by *LMNB1* and *LMNB2*, respectively. It seems that the mechanical stiffness of the nucleus is mainly dependent on A‐type lamins instead of B‐type lamins. The lamin A/C increases with the increase of matrix stiffness and impacts the differentiation of MSCs, whereas B‐type lamins remain relatively constant.^[^ ^98^, ^99^ ^]^ Meanwhile, the lamin A/C increases 30 folds from soft tissue (brain) to stiff tissue (bone).^[^ ^99^ ^]^ It is reported that lamin A/C deficiency in mouse embryonic fibroblasts can cause the absence of apical stress fibers and a lower cellular contractility.^[^ ^100^ ^]^ Partial silencing of lamin A/C in MSCs reduces myosin II contractility.^[^ ^101^ ^]^ Thus, the level of lamin A/C in the nucleus might represent the rigidity of the cells or tissues, reflecting surrounding matrix rigidity.^[^ ^101^ ^]^

Additionally, the highly contractile actomyosin filament bundles that cover a nuclear roof, known as actin cap, participate in regulating nuclear shape and mechanics.^[^ ^102^ ^]^ Actin‐cap fibers physically connect the apical surface of the interphase nucleus (through LINC complexes) to the basal surface of the cells (through actin‐cap‐associated focal adhesions, ACAFAs). This structure realizes enhanced tension on actin‐cap fibers^[^ ^103^ ^]^ through more activated myosin II than basal actin fibers.^[^ ^104^, ^105^ ^]^ For example, ACAFAs are more sensitive to changes in substrate compliance than conventional focal adhesions because they can bear higher tension than conventional basal stress fibers.^[^ ^100^ ^]^ Actin cap acts as a highway allowing the mechanical signal transmission between ECM and the nucleus. Especially, the actin caps have the apically polarized dorm structures, making them resistant to nuclear deformation.^[^ ^98^ ^]^ Meanwhile, the lamin A/C deficient cells display deformed nuclear morphology because they cannot form an actin cap. Therefore, disruption of the actin cap leads to anisotropic lamin A/C organization and a wrinkled nucleus.^[^ ^105^ ^]^

**Figure 5:**
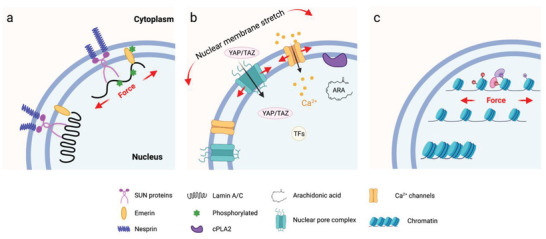
Nuclear membrane mechanotransduction. a) The nuclear envelope protein conformational changes responding to the exert force applied on the nucleus. b) Nuclear membrane stretch in response to force opens nuclear pore complexes, calcium channels, and activates cPLA2 on the cytoplasmic side, thus increasing calcium release, import of transcription factors (TFs), and production of arachidonic acid in the nucleoplasm. c) Mechanical forces applied to the nucleus may induce chromatin opening and epigenetic changes, that promote accessibility to TFs and regulate the downstream gene expression.

In response to the ECM microenvironment, cells tend to optimize their morphology to adapt to the complex microenvironment. This leads to physically extended or contracted nuclear pores, controls the transportation of molecules between cytoplasm and nucleus, and thus regulates gene expressions.^[^ ^106^ ^]^ For instance, the deformation of a nuclear membrane by physical cues can change the nuclear pore complex (NPC) permeability, unfolding of lamins, and activate lamins, SUN‐domain protein and emerin via phosphorylation (**Figure** **5**a).^[^ ^1^ ^]^ The stretched nuclear envelope facilitates the import of various transcriptional regulators such as Yes‐associated protein (YAP) and WW domain‐containing transcription regulator 1 (TAZ) into the nucleus and regulate gene expressions (Figure 5b). YAP/TAZ are essential sensors of external physical stimuli. The YAP/TAZ sense cytoskeletal tension and mediate cellular mechanoresponse via regulation of the focal adhesion kinase (FAK),^[^ ^107^ ^]^ Ras‐related GTPase RAP2,^[^ ^108^ ^]^ and the ARID1A‐containing SWI/SNF complex.^[^ ^109^ ^]^ On a stiff substrate, fibroblasts express more stress fibers and generate more tensions to flat the nuclear pores, leading to an increased import of YAP through NPC. While on a soft substrate, the nucleus is mechanically unconnected with the substrate, and the forces cannot transmit to the nucleus. Import and export of YAP are balanced between the internuclear and extranuclear environments (Figure 5b).^[^ ^110^ ^]^

Nucleus is used to be considered as a “hard disc” in cells that passively store the genetic information. Recent evidence has demonstrated their unique abilities to actively convert the external mechanical signaling into the biochemical signaling. Unlike the controversial cellular mechanosensing through the receptors within cell membranes, the plasma membrane, with its underlying cortical meshwork, directly “touches” the walls of confined spaces in vivo; it may sense the confinement. Lomakin et al.^[^ ^111^ ^]^ and Venturini et al.^[^ ^101^ ^]^ independently found that the nucleus of embryonic cells, immune cells (immature dendritic cells), and cancer cells (HeLa) can sense environmentally imposed confinement and respond to it. Briefly, when cell confinement is below the resting nucleus diameter, the nuclear undergoes deformation and unfolding, which leads to the nuclear membrane tension increase and the nuclear membrane stretch. The nuclear membrane then permits calcium release from internal membrane stores and activates the enzyme cytosolic phospholipase A2 (cPLA2), which triggers cell blebbing and specific contractile responses. This increases cell migration and enables cells to escape from narrow spaces (Figure 5b). Interestingly, recent studies have proved the direct effect of mechanical force on chromatin to manipulate transcription. Force can transmit through integrins, actin cytoskeleton, LINC complex, lamins, and direct stretch chromatin, resulting in an upregulation of transcription (Figure 5c).^[^ ^112^ ^]^ Overall, these stretch‐dependent transcription manners suggest that besides its original genetic functions, the nucleus can directly sense its physical microenvironments.

## 3 Mechanical Properties of Biomaterials Guide Stem Cell Fate

Besides the biological and biochemical cues, natural cell/tissue functions are highly impacted by the physical properties of microenvironments. These physical cues can be detected by cells and transformed into biochemical signals, regulating downstream cell activities. Understanding the mechanism of cellular activities and their interactions with materials at the genetic and molecular levels could significantly promote the development of biomaterials with controllable physical properties to trigger specific biological responses. It has been demonstrated that cellular interactions with the ECM are mainly based on the traction force generated by cells and surrounding materials through integrin‐mediated adhesions.^[^ ^46^ ^]^ Through the mechanotransduction process, cells sense the environment and modulate cell spreading, nuclear shape, intercellular signaling pathways, and traction forces, which in turn lead to the remolding of the microenvironment. This process is strongly dependent on the physical properties of the microenvironment, such as topography, spatial environment, elasticity, and other mechanical cues.^[^ ^113^ ^]^ These physical factors are highly mixed in living tissues, making the dramatically complex physical properties in vivo. Therefore, decoupling those factors appropriately has become a principal topic in mechanobiology. Hereby, we classify these physical properties of the biomaterials into the static cues (topography, elasticity, ligand presentation, etc.) and the dynamic cues (responsive cues and self‐regulated cues).

### 3.1 Static Cues

#### 3.1.1 Material Elasticity and Stiffness

**Figure 6:**
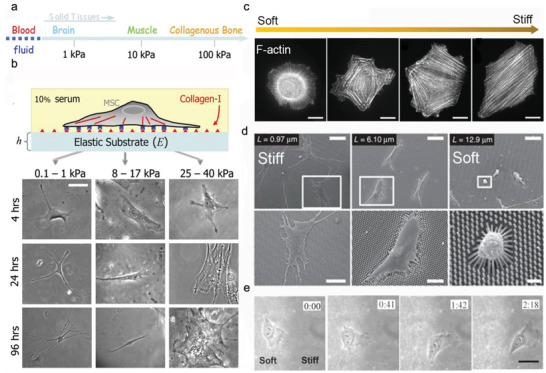
Substrate mechanics regulate cell adhesion and motions. a) The stiffness of different tissues. Reproduced with permission.^[^ ^119^ ^]^ Copyright 2006, Cell Press. b) Substrate elastic modulus regulates the differentiation of hMSCs. (Scale bar is 20 µm). Reproduced with permission.^[^ ^119^ ^]^ Copyright 2006, Cell Press. c) The substrate elastic modulus guides the actin cytoskeleton organization. Reproduced with permission.^[^ ^118^ ^]^ Copyright 2015, Nature Publishing Group. d) SEM images of hMSCs adhered on PDMS micropillar arrays of the indicated heights. Reproduced with permission.^[^ ^120^ ^]^ Copyright 2010, Nature Publishing Group. e) The movements of 3T3 cells on substrates with a rigidity gradient.^[^^]^ Reproduced with permission.^[^ ^121^ ^]^ Copyright 2000, Cell Press.

Adherent cells must adhere to a solid to realize their functions. The rigidity of the solid surface (also referred to as substrate stiffness), can be sensed by cells and influence cell behaviors. In human bodies, Young's modulus (the ratio of stress to strain referring to the elasticiy of materials) of tissues can vary by more than seven orders of magnitude, as low as 167 Pa for brain tissue and as high as 5.4 GPa for cortical bone.^[^ ^114^, ^115^ ^]^ This means that different types of cells prefer different grades of stiffness (**Figure** **6**a).

Various substrates with stiffness varying from a few hundred Pa to million Pa have been fabricated in a range of 2D/3D models by using natural materials such as alginate, chitosan, hyaluronic acid, agarose, and chitosan, as well as synthetic hydrogels such as polyethylene glycol (PEG), poly(vinyl alcohol) (PVA), and polyacrylamide (PAAm).^[^ ^116^, ^117^ ^]^ Cells can respond to substrate stiffness by altering their adhesion, spreading, cell phenotype, and migration characteristics. In general, stiff substrates could significantly facilitate cellular mechanoresponse and mechanosensing due to the increased intracellular tension balanced by the stiff substrate (Figure 6b). For instance, fibroblasts cultured on stiff substrates show a significantly larger spreading with denser actin stress fibers than those on soft ones. The orientations of actin filament display more aligned actin bundles on stiffer substrates (Figure 6c).^[^ ^118^ ^]^ Fu et al. fabricated a library of micro‐molded elastomeric micropillar arrays and utilized the heights to control substrate stiffness.^[^ ^120^ ^]^ The micropillar with a higher post possessed lower stiffness. The hMSCs spread well on the rigid surface with low‐height pillars (0.97 µm in height) but displayed a limited spreading on a soft array with high pillars (12.9 µm in height) (Figure 6d).

Cells can sense physical cues over relatively short distances, roughly the width of an adjacent cell.^[^ ^122^ ^]^ The cells could continuously respond to substrate gradient rigidity by adapting tissue geometry and exerting corresponding traction forces. This ability is called “durotaxis,” which has been implicated in many cellular processes.^[^ ^123^ ^]^ A gradient stiffness matrix leads cell migration toward the stiffer region that can offer stronger traction forces. For example, the 3T3 fibroblasts exhibit different polarities and orientations at the boundary of the soft and stiff areas. They can easily migrate across the border from the soft to the stiff site, resulting in a concurrent increase in cell spreading area and traction forces. Cells on the stiff region resist migrating to the soft side. They turn around or even retract when they reach the boundary from the stiff side (Figure 6e).^[^ ^121^ ^]^ Similar phenomena have also been reported in epithelial cells cultured on a microfabricated substrate engineered with gradient stiffness. The anisotropic rigidity induces the orientation of actin stress fibers or focal adhesions of epithelial cells and encourages their growth along the direction of the most significant rigidity.^[^ ^122^ ^]^ Interestingly, multicellular clusters also exhibit durotaxis, and the collective durotaxis is far more efficient than single cells. Cells coordinate their movements by actively interacting with each other, enabling rapid force transmission across cellular assemblies.^[^ ^124^ ^]^ The changes in the substrate mechanics modulate the cell–substrate adhesions, which in turn affect the cell–cell adhesions. This unique mechanical feedback may help us to understand many multicellular behaviors such as development, wound healing, and collective cancer cell invasions.^[^ ^125^ ^]^

The effect of substrate stiffness on stem cell phenotypes has been widely explored. The elastic property of substrates regulates gene expression and modulates transcription profiles, and therefore, it controls the lineage determination of stem cells (**Table** **1**). On 2D substrates, MSCs typically prefer osteogenic differentiation on stiff substrates, while preferring adipocytes or neurocytes on soft substrates. Polyacrylamide (PAAm) substrates have been fabricated to mimic the stiffness of different human tissues. On a soft substrate with a stiffness similar to the brain tissues (≈0.3 kPa), MSCs showed high expression of neuronal markers such as P‐NFH and *β*‐III tubulin. On a stiff substrate with 10 kPa that mimics muscle stiffness, stem cells expressed an enhanced myogenic marker (MyoD). While even more rigid materials (35 kPa) mimicking the collagenous bone caused the high expression of osteogenic marker Runx2.^[^ ^119^ ^]^ Interestingly, the proper stiffness condition has been suggested to be an effective strategy to maintain stem cells pluripotent. The mouse embryonic stem cells (ESCs) could maintain pluripotency up to 15 passages when cultured on a soft substrate (0.6 kPa). In contrast, these stem cells lost their self‐renewal and pluripotency under stiff culture conditions. It suggests that stem cells cultured on soft gels generate low cell‐matrix traction forces that facilitate the pluripotent maintenance of stem cells.^[^ ^126^ ^]^

**Table 1:** Static cues for mechanical regulation of cells

	Materials/technics	Molecular properties	Mechanisms and characteristics
Elasticity and stiffness	Hydrogels Membranes Pillars^[^ ^118^, ^119^, ^126^ ^]^	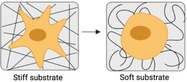	Affect the force loading rate of mechanical input on the integrin–receptor molecules interactions Coupled with other physical properties Complex in 3D culture environments
Topography	Lithography/pattern Surface roughening Material manipulation^[^ ^7^, ^142^, ^143^, ^144^ ^]^	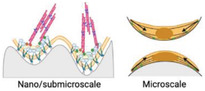	Size‐dependent cell mechanoresponse Microscale features reorganize the cellular cytoskeletal structures Nanotopography regulate the adhesion molecular structures Only for 2D culture
Geometry	Lithography/pattern^[^ ^145^, ^146^, ^147^, ^148^ ^]^	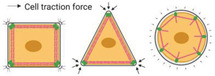	Geometry and size‐dependent cell mechanoresponse Reorganize cell actin filaments and cell tension
Ligand presentation	Lithography/pattern/chemical engineering^[^ ^149^, ^150^, ^151^, ^152^ ^]^	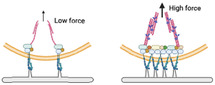	Ligand intensity affect the force loading rate of mechanical input on the integrin‐receptor molecules interactions Affect the adhesion molecules formation (integrin clustering, FAs)

As mentioned above, the nuclear shape is influenced by substrate stiffness. Lamin A is the nuclear sensor of substrate stiffness.^[^ ^99^ ^]^ When MSCs are cultured on a soft gel, the nuclear envelope appears with highly wrinkled morphology and low expression of lamin A. In contrast, the cells on stiff matrix express smooth nucleus and high expression of lamin A. The mass spectrum test indicates that lamin A in the cells grown on soft matrices experiences a higher phosphorylation process that promotes the disassembly and turnover of lamin A.^[^ ^98^, ^127^ ^]^

Besides, substrate stiffness affects the activity and distribution of certain nuclear transcription factors such as YAP/TAZ. A stiffer substrate has been demonstrated to promote nuclear flattening, thereby stretching nuclear pores, reducing resistance to molecular transportation, and promoting the YAP/TAZ nuclear localization.^[^ ^99^, ^110^ ^]^ YAP1 protein level decreases with the knockdown of lamin A, but it tends to translocate as expected into the nucleus with the increase of elasticity of substrates. Neither YAP1 transcript levels nor its binding partners or target genes change in lamin A knockdown cells. Interestingly, lamin A overexpression in cells on a stiff matrix could lead to a significant decrease in both the total YAP1 level and the nuclear localization, and YAP1 is observed to be enriched at the envelope of the nucleus. These results indicate that lamin A is not directly driving the expression or localization of YAP1 and vice versa.^[^ ^99^ ^]^

However, things become more complicated when it comes to soft hydrogel with unique physical or chemical properties. Other factors could also dominate the cellular mechanoresponse to soft materials. MSCs can readily attach and spread on soft elastomeric polydimethylsiloxane (PDMS) or silicone.^[^ ^128^ ^]^ But the similar results cannot be achieved on soft substrates such as polyacrylamide with the same elasticity and surface chemistry.^[^ ^129^ ^]^ One possible reason is that substrate surface energy and surface tension could regulate cell mechanosensing. Cheng et al. realized that the surface stress of silicones derived from surface energy dominates over their bulk elasticity on soft silicon that can be highly effective in activating cellular rigidity sensing pathways. Despite their intrinsic elasticity, all the silicon samples, such as biomedical silicones, liquid silicone oil, and model silicone gel, exert high surface stress that provides significant resistance to deformation induced by the cell tractions. This leads to the well‐spreading and comparable YAP and FAK activities of cells cultured on soft and stiff silicone surfaces.^[^ ^130^ ^]^ Interestingly, by incorporating the biocompatible surfactant (Span 85) into the soft silicone materials, the solid surface tension can be remarkably reduced and results in a decreased cell spreading area. The surface energy also alters the adsorption of ECM proteins.^[^ ^131^ ^]^ Snedeker et al. established collagen‐coated surfaces with varied surface energy using hydrophobic PDMS and hydrophilic polyethylene‐oxide‐PDMS (PEO‐PDMS), respectively. Although cell contractility was similarly diminished on soft substrates of both types, cell spreading and osteogenic differentiation occurred only on soft PDMS but not on hydrophilic PEO‐PDMS. They found that the collagen molecules deposited on the hydrophobic PDMS surface presented a folded conformation with prominent aggregates, which suggests a stronger collagen–collagen interaction than collagen–surface interaction. Meanwhile, the collagen deposited on the hydrophilic PEO‐PDMS surface displays an extended conformation, leading to a smooth monolayer collagen coating.^[^ ^126^ ^]^

Interestingly, hyaluronic acid (HA), the nonsulfated glycosaminoglycan polysaccharide in ECM, might alter cell response to matrix stiffness. Myocytes exhibited disorganized actin networks on soft (<1 kPa) polyacrylamide (PAA) gels. However, when HA replaced the PAA matrix as the gel network, a highly enhanced spreading area and myofibrillar assembly of myocytes occurred on soft substrates (200 Pa).^[^ ^132^ ^]^ HA, together with integrin ligands, promoted hepatocellular carcinoma cell spreading and migration on very soft substrates (300 Pa). The results were comparable to those grown on stiff substrates (30 kPa per glass) in the absence of HA.^[^ ^133^ ^]^ It has been found that cell interaction with HA can activate PI3K signaling pathway through an increase in P13K activity, which led to a higher level of PIP3 that promoted membrane tension to facilitate cell spreading without requiring high contractile forces from the substrates.^[^ ^133^ ^]^ We hypothesize that PI3K‐PIP3 signaling may activate Rac to promote actin polymerization, generating membrane tension. These findings provide a new strategy for the bioengineering of soft hybrid hydrogels.

Cells dynamically coordinate cellular machinery to generate force within the actin cytoskeleton and transmit through FAs.^[^ ^134^ ^]^ All these processes are energy demanded, and an increase in energy supply might enhance cell mechanosensing. Recent evidence suggests that the mechanoresponse on rigid substrates is initiated by cell spreading and the concomitant consumption of ATP to establish FAs and remodel the actomyosin network. Enhanced ATP production promotes actin cytoskeleton organization and FA formation, and increases cell spreading and tension. Genetic ablation of AMP‐activated protein kinase (AMPK) lowers cellular ATP level on a stiff substrate and strongly suppresses the cellular sensitivity to substrate stiffness.^[^ ^135^ ^]^

Most knowledge of stiffness‐dependent cell response in 2D microenvironments cannot be directly applied to 3D culture. In 2D culture, an increase in the matrix stiffness promotes MSCs spreading and proliferation. However, the MSCs encapsulated in a stiff, 3D hyaluronic acid hydrogel exhibit limited cell spreading and nuclear localization transcriptional factors activities.^[^ ^136^ ^]^ In natural 3D ECM, cellular tractions are distributed throughout the 3D space, propagated along with ECM, leading to the remodeling of the ECM and alteration of local mechanical properties.^[^ ^137^ ^]^ For instance, in 3D RGD‐modified alginate hydrogel, the osteogenesis of MSCs shows a biphasic response to the hydrogel stiffness, with maximal osteogenesis occurring at an intermediate stiffness, whereas 2D studies indicate a monophasic response for which the osteogenesis prefers the stiffer ones. By probing the cell‐ECM interaction via FRET, it has been found that the cells in the soft matrix tend to cluster the integrins by deforming the surrounding matrix. In contrast, this clustering is abrogated in the stiff 3D matrix.^[^ ^138^ ^]^ Furthermore, matrix degradation strongly influences cellular mechanosensing in 3D culture. The proper degradation of the cellular matrix increases cell‐mediated matrix remodeling, which in turn enhances cell dynamics and functions. An ideal scaffold degradation rate is that it matches cell growth and development. The 3D scaffolds often degrade too slowly or too fast. Even though many efforts have been made to establish cell‐compatible 3D networks for tissue engineering,^[^ ^125^, ^139^, ^140^, ^141^ ^]^ controlling the degradation kinetics remains a significant and persistent challenge in designing biomaterials.^[^ ^127^, ^142^, ^143^, ^144^ ^]^

#### 3.1.2 Surface Topographic Cues

The topography of materials is one of the critical factors that can regulate cell behaviors. Cells are embedded in ECM that possesses varied topographical features ranging from nanometers to micrometers. As the most abundant fibrous proteins within ECM, collagen molecules assemble into nano and microcollagen fibrils and fibers, inducing cell adhesion and polarization as well as promoting cell migration.^[^ ^153^, ^154^ ^]^ Besides ECM that provides abundant topographical cues, cells themselves are periodic and anisotropic. For example, muscle fibers are cylindrical multinucleated cells with diameters between 5 and 100 µm.^[^ ^155^ ^]^ In comparison, the cardiac tissues are consisting of highly patterned rectangular cardiomyocytes that are typically 100 to 150 µm in length and 20 to 35 µm in width.^[^ ^156^ ^]^

**Figure 7:**
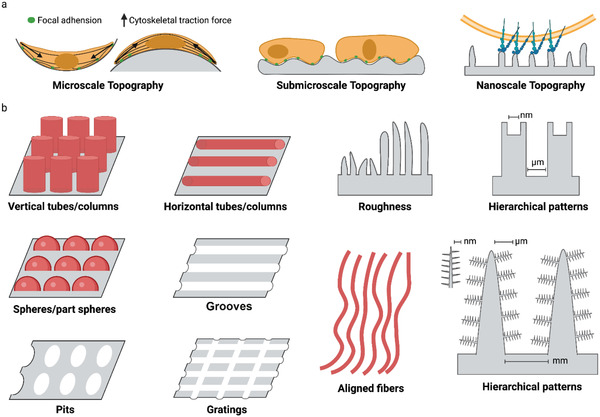
Surface topography affects cell mechanoresponse. a) Scale effect of topographic feature on cell adhesion. b) The factors affecting surface topography. These factors include roughness, anisotropic patterns (grooves, aligned fibers), and isotropic patterns (pillars/islands, pits, tubes/columns, and fibers).

Over the past decades, numerous biosurface topographies that contain microscale and nanoscale features such as roughness coatings, anisotropic patterns (grooves, aligned fibers), and isotropic patterns (pillars/islands, pits, tubes/columns, and fibers) have been constructed by using current nano/microfabrication technologies including photolithography, electron beam, self‐assemble, microcontact printing, replica casting, chemical etching, sandblasting, and electrospinning (**Figure** **7**b).^[^ ^113^, ^157^, ^158^, ^159^ ^]^ These surfaces recapitulate the topographical cues in the cell niche in a controllable and reproducible fashion, and afford a unique and powerful tool to reveal the mechano‐talk between cell and their surroundings.

According to the texture scale, surface roughness can be divided into macro/micro‐level roughness, submicro‐level roughness, and nano‐level roughness. Macro topography and microtopography generally provide cells with geometrical roughness cues ranging from microns up to millimeters, usually inducing cells to align with the anisotropy of the surrounding microenvironment—a phenomenon known as cellular contact guidance.^[^ ^158^ ^]^ Contact guidance has been suggested to affect cell polarization and actin cytoskeleton organization, thereby regulating various cell behaviors, including survival, motility, and differentiation (Figure 7A and Table 1). For instance, cell on convex surface, the curvature‐induced cytoskeletal tension may deform the nuclear, leading to a higher expression of lamin A and osteocalcin than that on concave surface.^[^ ^160^ ^]^ In a macroscopically parallel groove on titanium alloy (Ti6Al4V) surfaces with varied roughness (Ra: 0.30–1.8 µm), osteoblastic cells responded differently on the surface with different roughness.^[^ ^144^ ^]^ On a smoother texture (Ra: 0.50–0.60 µm), cells preferred to disperse anisotropically, showing a flattened, well‐spread appearance with a dense actin network. The increase in roughness brought higher ridges and lower valleys on the rough surface that may exert more substantial constraints. It enabled cells to elongate along the vertically oriented grooves, with more compact and less spreading thin actin filaments. In addition, cells grew slowly when the surface roughness exceeded the threshold (Ra = 1 µm). On the contrary, enhanced proliferation was observed in the cells grown on the surfaces with roughness ranging from 0.5 to 1 µm.^[^ ^161^ ^]^

Surface topographies at various scales have been applied in implant surface designs. For example, in bone regeneration, macro roughness improves the friction fit between the implant and bone to provide primary implant stability; micro‐roughness offers a larger surface area for bone cells to proliferate and deposit a newly formed bone matrix.^[^ ^162^, ^163^ ^]^

It is noted that cellular response is more complex at the nanoscale, where surface features are several orders of magnitude below that of cells (Figure 7a). At this scale, the size of surface features is similar to individual cell surface receptors (e.g., integrins). It may therefore be possible to target receptor‐driven pathways and manage the function of the cells including cell adhesion, differentiation, self‐renewal, and so on.^[^ ^164^ ^]^ The development of optimized micro/nano‐topographical ECM‐mimicking biomaterial surfaces has been considered as an excellent approach to enhancing cellular functions in tissue engineering and regenerative medicine.

Several studies have shown that surfaces with optimized micro‐and submicron‐scale physical characters enhance cell differentiation and local factor production in vitro. For instance, MSCs were cultured on hydroxyapatite discs with roughness ranging from 0.2 µm to 1.65 µm, and the osteogenic differentiation peaked at 0.7–1 µm.^[^ ^165^ ^]^ Polycaprolactone (PCL) surfaces with gradient roughness (0.5–4.7 µm) through the sand‐blasting process were generated to study the surface roughness on osteogenic differentiation of MSCs. As a result, the accumulation of osteogenic markers, including alkaline phosphatase (ALP), collagen type I, and mineralization, was found to be the largest on the 7 mm region of substrate with Ra of 2.1 µm.^[^ ^166^ ^]^

Physical nano‐roughness could also influence stem cell differentiation, but the conclusions are somewhat mixed. Dalby et al. found that poly(methyl methacrylate) (PMMA) surfaces with asymmetric and disordered nanopits (120 nm diameter, 100 nm deep, 300 nm spacing) stimulated MSCs to produce bone mineral in vitro without any osteogenic supplements.^[^ ^167^ ^]^ However, the MSC stemness was maintained for up to 8 weeks when cultured on polycaprolactone (PCL) surfaces embossed with regular square arrays of nanopits.^[^ ^168^ ^]^ The titanium surface with low roughness (15 nm in height) was more likely to promote the adhesion and osteogenesis of MSCs than the high ones with 55 to 100 nm in height.^[^ ^169^ ^]^ Whereas the other study indicated that the optimized roughness to do so was 150 and 450 nm rather than 20 nm on the same type of materials in another report.^[^ ^170^ ^]^ While no difference in cell adhesion on the titanium surfaces with different roughness was also reported.^[^ ^171^ ^]^

Such discrepancies can be explained by: 1) lack of a high‐throughput strategy to examine the effects with a broad range of roughness systemically. 2) complicated and time‐consuming fabrication protocol of rough surface and; 3) lack of mechanism understanding for cell mechanosensing to substrate roughness.

Our study fabricated a broad range of roughness gradient surfaces (Ra = 50 nm–1 µm) using a simple one‐step‐titled dip‐coating method.^[^ ^144^ ^]^ This easy‐to‐use coating allowed the systematic study of the cell mechanical response to different roughness. The cell mechanotransduction of hMSCs showed a biphasic manner and peaked at sub‐micron roughness region (Ra ≈ 280 nm), as characterized by the enhanced YAP nuclear localization and osteogenic differentiation in the peaked region. The nanoscale roughness enhanced cell adhesion and mechanotransduction via increasing specific surface area. Meanwhile, the cells on the microscale (Ra ≈ 1 µm) roughness regions preferred to adapt themselves to the confined surface structure by invading the microscale grooves, which suppressed the assembly of actomyosin cytoskeleton and the downstream cell spreading.^[^ ^144^ ^]^ Our findings highlight a tool for topographical gradient surfaces and offer a unique non‐invasive approach to exploring the interactions between cells and biomaterials.

Recently, the hierarchical surface, integrating both micro‐and nanoscale structures, has attracted intense interest in manipulating cell functions.^[^ ^172^ ^]^ Compared with the flat or simple nano‐ or microstructured surfaces, hierarchical micro‐/nano‐structure may provide more detailed topographic feature mimicking the native ECMs. Especially in bone renerations, the native bone structures are consist of complax nano‐, micro‐, and macroscale building blocks (e.g., osteon (ca. 100 µm), lamella (ca. 5 µm), fiber bundle (ca. 1 µm), mineralized fibril (ca. 100 nm) and nanophases (collagen molecules and mineral particles)).^[^ ^173^, ^174^, ^175^ ^]^ As a result, the nano/microhierarchical interfaces are more effective in modulating cellular response and inducing structural and functional integration of the cells and tissues.^[^ ^176^ ^]^ For example, Zreiqat et al. established a hierarchical strontium‐substituted hardystonite (Sr‐HT) ceramic coating integrated with the nanosized grains superimposed on the micron structure. Compared with the uncoated Ti‐alloy implants, this hierarchical structure highly enhanced new bone formation in animal experiments.^[^ ^177^ ^]^ Similarly, Ogawa et al. revealed that the nanoscale features (100–300 nm in diameter) at the hierarchical interface are more likely to promote stem cell differentiation and cell proliferation than on the microscale rough surface.^[^ ^178^ ^]^

Our recent study achieved a series of “raspberry”—like hierarchical surfaces to study the cellular mechanoresponse to the complex hierarchical features. The “raspberry” surface with well‐defined nanofeatures and tunable nano/microfeatures was prepared via the catecholic polymer coating technique. The smaller nanoparticles on the hierarchical surface provide more cell contact areas and enhance cellular mechanosensing by increasing the expression of filopodia and focal adhesions. Furthermore, the hierarchical interfacial characteristics could regulate the nuclear morphology and mechanics in a force‐depended manner via the tension of the actin cap. These studies highlight the significance of ECM‐mimicked nano/microhierarchical biointerfaces in regulating stem cell mechanotransduction, cell fate determination, and, more importantly, the structure size matter.^[^ ^142^ ^]^

**Figure 8:**
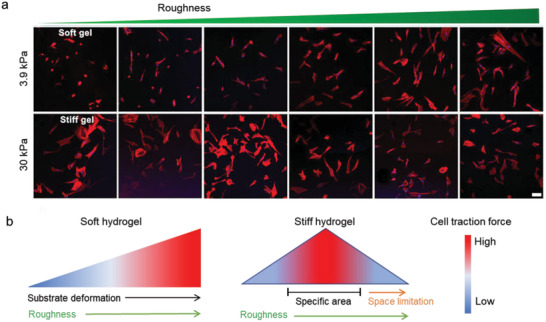
Cellular mechanoresponse to surface gradient roughness. a) The fluorescent images of MSC on roughness gradient soft and stiff hydrogels after cultured for 24 h. Scale bar inidicates 100 µm. b) Cells sense synergized roughness and stiffness stimulation.^[^ ^7^ ^]^ On the soft substrate, the high rough features provide more adaptable contact areas that allow the cell to largely deform the substrate, resulting in an enhanced mechanoresponse. In a stiff environment, the cell mechanoresponse shows a biphasic manner to the roughness features. The nanoscale roughness enhanced cell adhesion and mechanotransduction via increasing specific surface area. While the microscale features will limit the space for cell extension, suppressing cell adhesion and tension.

So far, most of the studies have been focused on topology itself. Most of the reported material substrates exhibit stiffness ranging from MPa to GPa, far exceeding the stiffness sensing by cells in vivo (from a few Pa to hundreds of kPa). It is challenging and necessary to establish the patterned substrates combined with other physical cues, especially stiffness. We recently developed stiffness‐controllable hydrogels with a wide‐scale surface roughness gradient (Ra = 200 nm–1.2 µm) by soft lithography. MSCs could sense and respond to surface topographic features in a stiffness‐dependent manner. Specifically, the high surface roughness (Ra ≈ 1 µm) enhanced cellular mechanotransduction on very soft substrates (3.8 kPa), which was comparable to that on smooth, stiff ones (**Figure** **8**a). Meanwhile, compared to the soft and smooth surface, the cells largely deformed the soft but rough substrates to reshape the adhesive environment. It may ascribe to the more binding sites and lower stiffness provided by the highly rough features. Our study suggests that the deformable soft substrate can change local mechanical properties by reorganizing the density/structure of polymer networks induced by force, thereby enhancing integrin‐clustering and cellular mechanotransduction (Figure 8b).^[^ ^7^ ^]^ A similar phenomenon has been observed in soft fibrillar microenvironments. Baker et al. found that fibers with lower stiffness were more easily deformed by force transmitted from nearby fibers. It led to an increased ligand density at the local adhesion sites, promoting focal adhesion and signaling formations.^[^ ^21^ ^]^ Interestingly, these resulting curved fiber networks have recently been found to promote cell bridge formation due to the condensed actomyosin filaments near the curved edge of cells. It enabled cells to generate higher myosin‐based intracellular force than the straight fibers.^[^ ^160^ ^]^

The shape and structure of nuclear are strongly affected by nano and microtopography. MSCs were cultured on micropillar poly(lactide‐co‐glycolide) (PLGA) arrays. The deformation of the nucleus was initiated on the micropillar substrate with a height of 3.2 µm, and raised to the maximum when the micropillar height increased to 4.6 µm or larger).^[^ ^179^ ^]^ Further study indicates that the nuclear deformation of cells in confined space is regulated by actomyosin‐based contractility coupled with the LINC complex.^[^ ^180^, ^181^ ^]^ Unexpectedly, the micropillar arrays can induce nuclear deformation but with limited spreading areas. Still, it can induce enhanced osteogenesis and attenuated adipogenesis of the MSCs.^[^ ^179^ ^]^ Even the underlying mechanisms remain unclear. It is possible that chromosomal territories repositioning caused by significant self‐deformation of cell nuclei alters gene expressions and ultimately influences the differentiation potential of the cells.^[^ ^143^ ^]^

#### 3.1.3 Geometrical Cues

**Figure 9:**
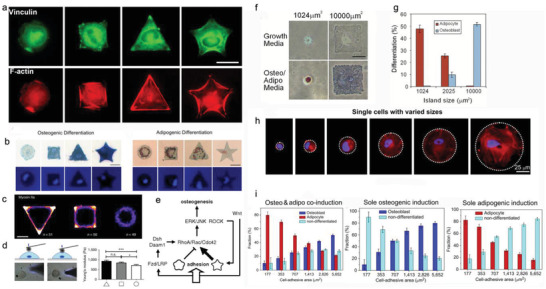
Effect of cell geometry and cell spreading area on cell adhesion, mechanics, and differentiation. a) The fluorescent images of single cells on microislands with different shapes. Green: vinculin, red: actin. Scale bar indicates 25 µm. Reproduced with permission.^[^ ^146^ ^]^ Copyright 2011, Elsevier. b) Osteogenic and adipogenic differentiation of stem cells on microislands with indicated different shapes. ALP was stained in blue. Lipids were stained in red. (Scale bar: 25 µm). Reproduced with permission.^[^ ^146^ ^]^ Copyright 2011, Elsevier. c) Heat maps representation of the of myosin IIa expression for cells on microislands with different shapes. Scale bar indicates 20 µm. d) Precise measurement of mechanical properties of live‐cell by atomic force microscopy. Reproduced with permission.^[^ ^145^ ^]^ Copyright 2018, Nature Publishing Group. e) Speculative pathway for shape‐directed differentiation of adherent cells. Reproduced with permission.^[^ ^147^ ^]^ Copyright 2018, National Academy of Sciences. f) The bright‐field images of differentiated hMSCs on rectangular fibronectin‐coated islands with different areas. ALP was stained in blue (osteogenesis). Lipids were stained in red (adipogenesis). Scale bar indicates 25 µm. Reproduced with permission.^[^ ^145^ ^]^ Copyright 2018, Nature Publishing Group. g) Quantification of differentiation of hMSCs cultured on the microislands with different areas after one week of induction. Reproduced with permission.^[^ ^145^ ^]^ Copyright 2018, Nature Publishing Group. h) Fluorescence images of single hMSCs cultured on microislands of varied areas. Red: F‐actin, blue: DAPI. Reproduced with permission.^[^ ^148^ ^]^ Copyright 2012, Elsevier. i) Quantification of differentiated cells in different cell induction conditions. Reproduced with permission.^[^ ^148^ ^]^ Copyright 2012, Elsevier.

Cells in the body are confined by neighboring cells and ECM, which provide them with geometrical cues to sense and respond.^[^ ^182^ ^]^ Even the exact mechanisms remain unresolved, cells can translate physical geometry into cell geometry that affects various cellular processes such as cell survival, proliferation, and differentiation (Table 1).^[^ ^183^ ^]^ A series of isotropic microislands with circular, square, triangular, and star shapes have been successfully fabricated by transfer lithography, and the microisland area has been adjusted for single‐cell adhesion. MSCs attached to these islands spread in the same shape as the underlying islands. With the increase of shape angles of microisland, MSCs generated larger tension, and exhibited larger areas of FAs and denser actin bundles (**Figure** **9**a), showing shape‐dependent cell contractility (Figure 9c), and cell stiffness (Figure 9d).^[^ ^145^ ^]^ Correspondingly, osteogenesis and adipogenesis were enhanced in star cells and round cells, respectively (Figure 9b).^[^ ^146^, ^184^ ^]^ Consistently, with the increase in the aspect ratio of rectangles or the rise in the curvature of pentagonal symmetry, the osteogenesis of MSCs was enhanced, but the adipogenesis was decreased.^[^ ^147^ ^]^ Further study indicates that cell geometry can regulate plasma membrane order via controlling the abundance of lipid rafts and caveolae, which modulates stem cell fate through Akt signaling pathways.^[^ ^145^ ^]^ On the other hand, MSCs exerted larger cell contractility on polygon microisland by activating tension‐specific MAP kinases (p38, ERK1/2, JNK) and promoting Wnt signaling,^[^ ^147^ ^]^ and subsequently led to osteoblast differentiation (Figure 9e).^[^ ^185^, ^186^ ^]^

The physical geometric cues could control cell spreading area and determine cell fate. Chen et al. fabricated fibronectin‐coated round and square islands of different sizes via the soft lithography technique. The rest regions were coated with non‐adhesive polymer pluronic F08.^[^ ^187^ ^]^ After 7 d of incubation, MSCs on the largest (10 000 µm^2^) island were able to flatten and differentiate into osteoblasts via activating RhoA. In contrast, cells with the smallest area (1024 µm^2^) displayed a round morphology and adipogenic differentiation induced by dominant‐negative RhoA. (Figure 9f,g). Further, a linear change in osteogenesis and adipogenesis was achieved by orchestrating island areas (Figure 9h,i).^[^ ^148^ ^]^ Generally, a large spreading area enhances cytoskeletal tension, which activates ROCK and RhoA and results in the osteogenesis of MSCs.

**Figure 10:**
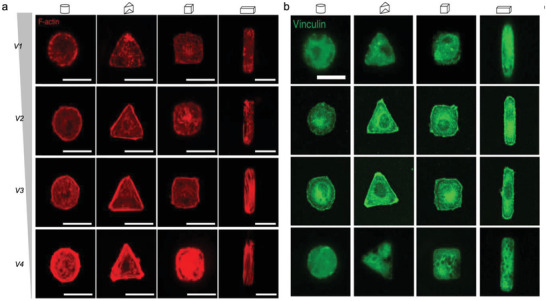
Effect of cell volume and geometry on cell adhesion and mechanics. a) The actin and b)vinculin expression of cells on microinches with distinct geometry and volumes. Green: vinculin, red: F‐actin. Scale bar for all images indicates 20 µm. Reproduced with permission.^[^ ^182^ ^]^ Copyright 2017, Nature Publishing Group.

3D micropatterned systems provide cells homogeneous microenvironment of defined volume, and cells can form adhesive connections on all sides.^[^ ^15^ ^]^ Both geometry and volume of 3D microniches strongly influence cell function. Consistent with the findings on 2D patterns, the 3D niches with more shape angles or increased aspect of ratios promote FA formation and cell tension generation. For instance, MSCs in triangular prism and cuboid show markedly denser stress fibers and enhanced FAs than those cultured in cylinder and cube (**Figure** **10**a). However, F‐actin organization and FAs are not sensitive to the cell shape (triangular or cylinder) in cells with greater (V1) or smaller volumes (V4) (Figure 10a,b), suggesting the close correlation between the volume of 3D microniche and cell functions. The decreased cell volume induces increased stress fibers, FA formation, and cell tension in a specific scope, which further affects nuclear mechanoresponse and cell phenotype. It has been considered that cell volume changes mRNA concentrations and thus leads to different interactions between key regulatory proteins. Cells with large volumes are found with diluted mRNA concentration and decreased RhoA, Arp2/3, TEAD transcripts that play central roles in actin fiber formation. This leads to a much less pronounced actin cytoskeleton organization and downstream biochemical signaling.^[^ ^182^ ^]^

The geometric cues can be translated into cellular physical force signals that alter nuclear architecture.^[^ ^188^ ^]^ A series of fibronectin‐coated rectangular microislands with the same area (1600 µm^2^) but different aspect ratios have been developed to study how cell shape regulates nuclear shape.^[^ ^189^ ^]^ It is found that the deformation and orientation of the nucleus occur as the cell elongated, controlled by lateral compressive forces applied by central thick stress fibers on both sides of the nucleus, and vertical compressive forces exerted by apical actin filaments constrains nuclear height. As a result, cell elongation triggers nuclear elongation along the longer axis of the cell body, leading to extreme chromatin condensation and decreased cell proliferation.^[^ ^189^ ^]^ It should be noticed that tension in lateral stress fibers is strongly dependent on FA formation. At the same time, the FA area increases with the extent of cell elongation, confirming that cell elongation increases cell tension and subsequent nuclear deformation. Recently, the process of how cell geometry impacts local tensile stresses and subsequent feedback of cytoskeleton and nucleus has been simulated by a 3D chemomechanical model. For substrates with high aspect ratio, the initial contractility of the cell is generated particularly at cell boundaries, the adhesion molecules experience higher tensile stresses at the front and rear of the cell, followed by an increase of stiffness and mature FAs at the two ends. The local tensile stresses generated at the mature FAs activate mechanotransduction, leading to the increase of the actin filament network and actomyosin contractility along the direction of the tensile stresses. With the assistance of ACAFAs, the nuclear envelope is imposed by significant vertical and lateral compressive forces, leading to a flattened and elongated nuclear morphology. Nuclear accumulation of HDAC3 and condensation of chromatin occur as the shrinkage of the nuclear volume. In contrast, the nucleus on the circular substrate experiences lower and isotropic tension, resulting in a round morphology and lower nuclear stiffness.^[^ ^190^ ^]^

#### 3.1.4 Interfacial Ligand Presentation

**Figure 11:**
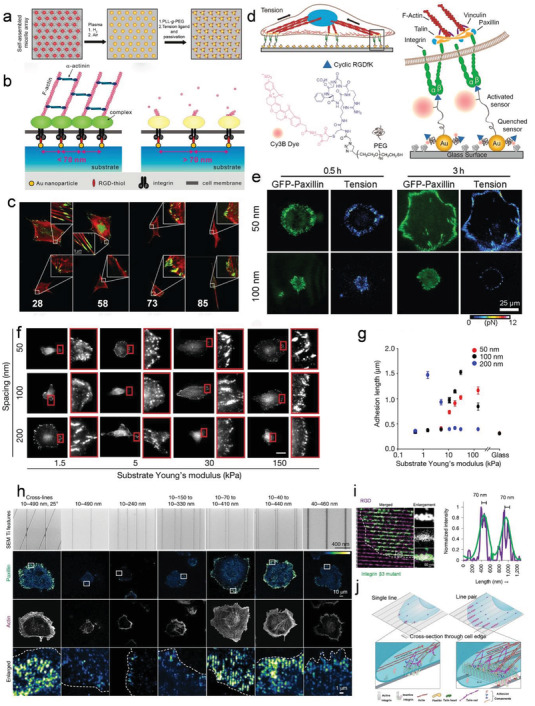
Nanopatterned adhesive interfaces regulate cell adhesions via activating integrin. a) The fabrication process of BCMN patterned nanoarray for cell adhesion. Reproduced with permission.^[^ ^154^ ^]^ Copyright 2012, Rockefeller University Press. b) Ligand spacing influences the FAs formation. Reproduced with permission.^[^ ^151^ ^]^ Copyright 2009, American Chemical Society. c) Cells adhere to the Au‐nanodot patterned surfaces with (up) and without (down) c (RGDfK). Reproduced with permission.^[^ ^151^ ^]^ Copyright 2009, American Chemical Society. d) Illustration showing the working principle and chemical structure of the molecular force probes on the AuNP patterned substrate. e) Integrin tension, cell shape, and FAs were monitored for cells adhered on the AuNP patterned substrate at different time points. Reproduced with permission.^[^ ^150^ ^]^ Copyright 2014, American Chemical Societys. f) Force loading rate explains spatial sensing of ligands by cells. Cells seeded on polyacrylamide hydrogel with different rigidities and nanodot spacings. Reproduced with permission.^[^ ^75^ ^]^ Copyright 2009, AAAS. g) Quantification of focal‐adhesion length for cells on the substrate with varied rigidities and ligands spacing. Reproduced with permission.^[^ ^74^ ^]^ Copyright 2017, Nature Publishing Group. h) Representative images of cell adhesion on nanoline array with different spatial properties. Red (actin, phalloidin), green (paxillin). The numbers indicated the detailed geometric parameters of nanolines. The first number represents the width of lines, the second number represents the internal distance between the two lines in pair, and the third number indicates the space between adjacent line pairs. Reproduced with permission.^[^ ^152^ ^]^ Copyright 2019, American Chemical Society. i) The mutant *β*3 integrins bind to nanolines functionalized with the RGD ligands. Line plot across cluster region showing mutant *β*3 (green) and the line pair (magenta). Reproduced with permission.^[^ ^152^ ^]^ Copyright 2019, American Chemical Society. j) The proposed models for assembling adhesion nanoclusters on single nanoline and paired nanolines. Reproduced with permission.^[^ ^149^ ^]^ Copyright 2019, Nature Publishing Group.

Cells can sense the density and distribution of ECM ligands via individual integrin proteins and integrin‐based transmembrane complex.^[^ ^6^ ^]^ Thus, ligand presentation could modulate cell behaviors, such as ligand concentration and spatial distribution (Table 1). Landmark 2D nanopattern technologies have been developed to realize the precise control of the spatial distribution of ligands. By applying block or diblock copolymer micelle nanolithography technology (BCML), Spatz and co‐workers have realized nanopatterning of ligands such as RGD on a 2D surface.^[^ ^191^ ^]^ Briefly, glass slides are patterned with regulated gold nanodots, and each gold nanodot is functionalized with linker molecules. The nanodots are then transferred to a nonadhesive hydrogel via the linkers and grafted with cell adhesive ligands such as RGD (**Figure** **11**a). The matching of nanodot (about 10 nm) and integrin (8–12 nm) diameters^[^ ^191^, ^192^, ^193^ ^]^ achieves a one‐to‐one correspondence between a nanodot and an integrin receptor.^[^ ^194^ ^]^ It thus enables to control the spatial distribution of cell integrins.

The space between the nanodots drastically affects the formation of focal adhesions. Spatz et al. and Ding et al. initially indicated that an inter‐ligand spacing of less than 70 nm on stiff or rigid surfaces was essential for successful integrin clustering and cell spreading.^[^ ^189^, ^193^, ^195^ ^]^ They found that many adherent cells, such as osteoblasts, fibroblasts, and melanocytes, could adhere and spread well on the patterned surfaces with interligand space of 28 or 58 nm. However, when the ligands space was larger than 73 nm, FA formation collapsed, even though the cells were still bound on the surface. These led to a ruffling cell membrane and significantly altered cell polarity and morphology (Figure 11b). It may ascribe that adhesion‐related proteins such as talin and *α*‐actinin cannot bridge integrins that are too far away to form stable integrin clusters.^[^ ^191^, ^196^ ^]^ It is noteworthy that integrin clusters require small ligand space in 2D for stabilization.^[^ ^149^, ^197^ ^]^ Similar phenomenon has been observed on nano‐lines. Sheetz group fabricated RGD functionalized nanolines of titanium (Ti) or gold‐palladium (AuPd) by electron‐beam lithography, mimicking 1D (single thin lines with width ≤30 nm) or 2D geometries (single wide lines width >40 nm, crossing lines, or paired lines) of ECM fibers (Figure 11h).^[^ ^149^ ^]^ Single thin lines did not support cell spreading, but wide lines did. Dense integrin clusters formed when parallel lines were closely spaced (<110 nm) or crossed by recruiting activated but unliganded integrins (Figure 11i,j).^[^ ^149^ ^]^ Thus, a 2D area (>40 nm) is needed to support the force‐dependent maturation of adhesions.

To reveal how integrin ligand clustering affects the integrin mechanosensing, molecular tension fluorescence microscopy has been employed to monitor the integrin tension dynamics during focal adhesion formations (Figure 11d).^[^ ^150^, ^151^ ^]^ In the initial nascent adhesion formation stage, actin polymerization contributes the integrin tension, which is at an average of 1–3 pN on the nanoarrays with 50 and 100 nm distance. During the FA maturation process, critical ligand spacing (<60–70 nm) enables bound integrins to harness actomyosin‐driven tension to increase their average tension to ≈6–8 pN, which fascinates and stabilizes FA formation. The average tension markedly decreases with ligand spacing above 100 nm due to the destabilized integrin clusters (Figure 11e,f). These quantified integrin tension dynamics are perfectly in line with the previous cell adhesion results, which are essential to revealing the force transmission through integrin‐based adhesions in mechanotransduction.

The cell migration and polarity on the substrate with gradient nanoparticles spacing have been further explored. A BCML patterned surface with ligand spacing gradients varying from 50 nm to 250 nm was fabricated. Osteoblast cells at around 80 nm distanced nanodots preferred to migrate to the region with denser ligands with space about 60–70 nm and polarized in parallel with the same gradient direction. Interestingly, cells show extremely high sensitivity to the spacing gradient. The weakest gradient that cells can sense can be as low as ≈15 nm mm^−1^. Cells can respond to the minimum difference in ligand patch spacing to ≈1 nm between the front and rear of the cell.^[^ ^198^ ^]^

At the micro/nanoscale, the integrin ligands presented in the ECM are not distributed continuously or uniformly. Cell adhesions are different in the cells cultured on 2D substrates with uniform or anisotropic‐coated ligands. The ordered ligand nanopatterns on 2D substrates with a ligand spacing of >70 nm highly restrict cell spreading. However, a disordered nanopattern with a global average inter‐ligand spacing of >70 nm enables clustering integrins. The disordered pattern provides polydispersity of local interligand spacing, and stable adhesions could form on the area where the inter‐ligand spacing < 70 nm.^[^ ^151^ ^]^ Mrksich group used polymer pen lithography (PPL) technology to generate nanoscale adhesive patterns with anisotropic geometrical features. The anisotropic focal adhesions around the periphery of symmetric patterns promoted the contractile actin cytoskeleton. Even seeded on a circular substrate, the anisotropic ligands facilitated increased cell contractility and redirected stem cell differentiation from adipogenesis to osteogenesis.^[^ ^152^ ^]^ Bian et al. fabricated RGD‐bearing gold nanorods (AuNRs) with different aspect ratios (ARs, from 1 to 7) for nanoscale anisotropic ligand presentation. AuNRs with larger ARs dramatically enhanced cell spreading compared to the smaller ones. The AuNRs with large ARs facilitated the recruitment of both *β*1 and *β*3 class integrins, which promoted the formation of mature FA toward fibrillar adhesion and activated mechanotransduction signaling molecules. Hence, the anisotropic presentation of ligands by large AR AuNRs promoted stem cell osteogenesis both in vitro and in vivo.^[^ ^199^ ^]^ Recently, the Kang group developed a heterogeneous cylinder nano‐barcoding system consisting of RGD‐bearing Au and RGD‐free Fe nano‐segments. The total length of Fe or RGD‐Au was kept constant in all groups. Compared to those frequently disconnected RGD‐Au segments (with an adhesive length of 30 or 75 nm), the barcodes with more continued RGD‐Au segments (150 nm or 300 nm) facilitated focal adhesion and mechanosensing of hMSCs. They thus promoted their osteogenic differentiation both in vitro and in vivo.^[^ ^150^ ^]^ It is also interesting that terminally positioned RGD‐Au instead of internally positioned ligands enhances cell adhesion,^[^ ^200^ ^]^ and the underlying mechanism remains to be elucidated.

The mechanism of cellular response to ligand spacing is still under debate. We discussed that ligand distance and geometry could regulate the adhesion cluster formation in adhesive dot‐ and line‐based models. However, the transient nature of the integrins aggregated on large spaced dots or single lines could not support integrin cluster formations. Large FAs form when cells are seeded on nanopatterned substrate with ligand spacing with ≈70 nm or paired lines separated with ≈110 nm.^[^ ^149^, ^151^ ^]^ The integrin nanocluster bridges are especially found on the paired line models composed of ligand‐bound and unliganded integrins. These adhesion clusters subsequently assemble into stable, dense focal adhesion. This indicates that integrin clustering and 2D ligand geometry are required for cellular mechanosensing.

The other view is that cells do not sense ligand spacing directly; instead, they sense the ligand density through individual integrin—ECM bonds—the molecular clutches. The traction force loading on each molecular clutch is critical for further recruitment of extra integrins. The increase in ligand spacing would decrease the number of molecular clutches. Thus, the force exerted by myosin would be distributed among fewer clutches, leading to an increased force loading on each clutch. Roca‐Cusachs and co‐workers developed hydrogels with tunable rigidity (1.5–150 kPa) and various spaced adhesive ligands (50, 100, 200 nm).^[^ ^74^ ^]^ Focal adhesions were enhanced with the increase of ligand spacing on hydrogels with low rigidity (1.5 kPa, 200 nm), but collapsed on stiff hydrogel (150 kPa, 200 nm) (Figure 11f,g). On soft hydrogel (<1 kPa), the force loading rate was too low to reach the force threshold for focal adhesion formation. However, as rigidity increased (1.5–30 kPa), clutches with higher spacing were more likely to reach the force threshold, promoting integrin recruitment. On substrate with high rigidity, excessive force loading at integrin–ECM bonds could not be compensated with adhesion growth, leading to the collapse of focal adhesion. This study reveals that cells sense spatial and physical information at the nanoscale by regulating molecular force loading. This process is impacted by substrate rigidity, ligand distribution, and contractility. Probably, the means of cellular response to ligand spacing is stiffness dependent.

Accordingly, ligand distance‐guided cellular fate determination is dependent on substrate rigidity. MSC mechanosensing and osteogenesis are enhanced on soft hydrogels (ca. 3 kPa) but suppressed on stiff hydrogels (ca. 40 kPa) with the increase of ligand spacing (30–230 nm).^[^ ^8^ ^]^ However, Ding et al. reported ligand spacing affects the stem cell spreading and differentiation unconventionally. Large ligand spacing (135 nm) significantly limits cell adhesion but promotes osteogenic differentiation compared to small ligand spacing (49 nm) on the remarkably stiff (130 kPa and 3170 kPa) substrates.^[^ ^201^ ^]^ We confirmed these findings and found that YAP/TAZ are highly concentrated in cell nucleus on the very stiff and rigid hydrogels with large ligand spacing despite the limited cell adhesions. The intracellular force is above the YAP/TAZ nuclear localization threshold even when the ligand spacing is large. The cell osteogenic differentiation is initiated by an exciting mechanism (unpublished results). Moreover, on a stiff substrate with nanohole patterns, osteogenic differentiation of hMSCs is enhanced on the substrate with a smaller inter‐integrin distance (34 nm). In comparison, the adipogenesis prefers a larger integrin space (62 nm).^[^ ^202^ ^]^ In addition, the matrix of specific periodicity of 63 nm helical ribbon shape could promote stem cells commitment into osteoblast lineage, whereas no osteoblast commitment is observed when the binding site distance increased to 100 nm on twisted nanoribbons.^[^ ^203^ ^]^ The increased ligand spacing inhibits the clustering of integrins, which results in the disassembly of FA and stress fiber. It further leads to low actomyosin contractility and poor activity of RhoA signaling.^[^ ^81^, ^204^ ^]^

### 3.2 Dynamic Cues

The natural ECM is highly complex and dynamic that undergoes constant remodeling to regulate cell behaviors and functions in vivo.^[^ ^9^ ^]^ The structures and properties of ECM change over time through variations in composition, reorganization of the macromolecular networks, and enzyme‐mediated degradation.^[^ ^10^ ^]^ Both the spatial arrangement and timed presentation of these cues matter in the regulation of cell behaviors and fate. Therefore, the static mechanical properties such as chemistry, topography, and stiffness could not address challenges in biomaterials and healthcare studies. To improve the understanding of how cells sense and respond to microenvironment and how these dynamic cues govern cell functions, the dynamic elements need to be incorporated within the biointerface. So far, only very limited types of biomaterials with dynamic mechanical properties have been generated. The influence of their features on cellular behaviors has been progressively elucidated. According to the ways of mechanical stimulation applied to the cells, the dynamic mechanical interactions can be sorted into stimuli‐responsive and self‐regulated cues.

#### 3.2.1 Stimuli‐Responsive Cues

To study cell response against dynamic behavior of ECM, significant efforts have been made to develop novel stimulus‐responsive bio‐interfaces. These dynamic bio‐interfaces are commonly composed of ECM molecules and stimuli‐responsive motifs. The stimulus, such as temperature, light, electrochemistry, magnetic force, mechanical stretching, and biomolecules, have been employed to trigger continuous changes in either chemical or physical interface properties.^[^ ^10^ ^]^ Most of these materials can only switch between two conditions, and the cells sense the static cues in each condition. This type is quasi‐dynamic, which is briefly introduced in this review. The other type can provide continuous physical or chemical stimulations for cells as dynamic systems. The current stimuli‐responsively dynamic materials and mechanisms are summarized in **Table** **2**.

**Table 2:** Stimuli‐responsive materials for dynamic regulation of cells

Stimulus	Responsive molecules	Mechanism	Characteristic
Temperature	PNIPAm	Apply tension or compression to cells via swelling or shrinkage^[^ ^205^ ^]^	Reversible
	Sharp memory polymer	Topography change^[^ ^206^, ^207^ ^]^	
Light	Azobenzene	Control the presentation of cell adhesive ligands by isomerization^[^ ^208^, ^209^ ^]^	Spatially controllable Easily functionalized Potential biotoxicity (UV)
	Nitrospiropyran	Control the presentation of cell adhesive ligands by isomerization^[^ ^210^, ^211^ ^]^	
	*O‐*Nitrobenzyl derivatives	Change the stiffness by cleavage^[^ ^207^ ^]^ Control release of the tethered biomolecules^[^ ^212^ ^]^ Control the presentation of cell adhesive ligands by cleavage^[^ ^213^, ^214^ ^]^	
	Photoinitiator	Change the pore size and gel stiffness by polymerization^[^ ^215^ ^]^	
	Proteins	Light exposure disassembles/assembles the protein structures^[^ ^216^ ^]^	Limited adjusting range of hydrogel mechanics
Electric field	Hydroquinone	Conformational change of molecules by oxidation^[^ ^217^, ^218^ ^]^	Rely on conducting substrates
Mechanical force	PDMS/PNAGA	Apply tension or compression to the cells^[^ ^219^ ^]^ Topography change^[^ ^220^ ^]^	Reversible Topography control No spatial control Only for 2D culture
Magnetic force	Magnetic nanoparticles	Apply tension or compression to cells or regulate the cell adhesive ligand mobility^[^ ^221^, ^222^ ^]^	Reversible Spatially control
Biomolecules	Enzyme	Change the substrate stiffness or release of tethered biomolecules^[^ ^223^ ^]^	Highly specific Nonreversible
Ions	Protein	Ions trigger protein binding^[^ ^223^ ^]^	Highly specific Limited adjusting range of hydrogel mechanics

##### Temperature Stimulation

**Figure 12:**
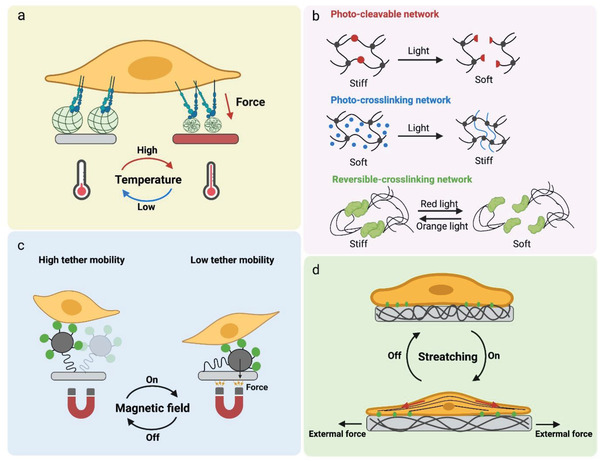
Stimuli‐responsive materials for dynamic regulation of cell behaviors. a) Thermoresponsive materials have been utilized to design optomechanical actuator nanoparticles that are regulated by temperature to control receptor tension. When the temperature is lower than the LCST, the materials will collapse and exert force to the cell. b) Photoresponsive materials regulate the mechanics of the cellular microenvironments by cleavage/unbinding or crosslinking/binding of polymer/protein networks. c) Magnetic force regulates the cell ligands' binding force and kinetics. d) The external mechanical stretching can induce a controllable reinforcement and reorientations of stress fibers, thereby regulating the cell mechanotransduction.

Thermoresponsive materials that undergo apparent variation in their physiochemical property in response to temperature changes have been widely applied in the development of dynamic matrix. The temperature changes usually cause changes in the hydration state of the materials, resulting in wettability and morphology changes in the matrix. Poly(N‐isopropylacrylamide) (PNIPAAm) is one of the most popular materials used in thermo‐activatable systems.^[^ ^224^ ^]^ The lower critical solution temperature (LCST) of PNIPAAm is 32 °C, which means PNIPAAm is hydrophobic over 32 °C and changes to a hydrophilic state below 32 °C. This property allows the application of PNIPAAm in cell sheet engineering that can be directly used for therapeutic purposes.^[^ ^225^ ^]^ Specifically, the cells can grow on PNIPAAm functional surfaces at 37 °C, and detach from the underlying ECM proteins below 32 °C (**Figure** **12**a).^[^ ^226^ ^]^

These thermo‐responsive materials have been utilized to design optomechanical actuator nanomaterials or hydrogel that are regulated by near‐infrared (NIR) light to control receptor tension.^[^ ^227^, ^228^, ^229^ ^]^ These technics provide a spatially selected force stimuli for single cell manipulation. Specifically, PNIPAAm was coated on Au nanorods and functionalized with cell surface receptor ligands. The Au nanorod can convert the NIR light to localized heat that drove polymer collapse. The particle collapse exerted a force per ligand of 13–50 pN. With the NIR stimulation at different frequencies, these nanoparticles could provide cyclic or sustained mechanical signals to target cell surface receptors. It was found that cyclic pN force stimulation instead of sustained force would strain the integrin receptors and activate mechanosensitive proteins such as talin and vinculin, leading to FA maturation and actin polymerization. Meanwhile, the pN forces could be delivered to the target area of the cell edge by illumination, to manipulate FA formation, cell protrusion, and migration.^[^ ^229^ ^]^

Unlike the traditional static substrates with defined topography, the thermoresponsive shape memory polymers (SMP) could achieve programmed changes in the surface topography during cell culture.^[^ ^206^ ^]^ They can change from a temporary shape to a memorized permanent shape upon a particular trigger, such as electrical, thermal, or solvent activation.^[^ ^230^ ^]^ Henderson et al. developed a polycaprolactone (PCL) based SMP surface to study cell behavior on dynamic surface nanopatterns.^[^ ^231^ ^]^ The initially flat SMP substrate was embossed to exhibit a temporary topography of microgrooves. The cells seeded in this grooved substrate spread align with the orientation of the parallel grooves at room temperature. When the temperature exceeded 37 °C, the substrate was transformed to its original smooth state. Accordingly, these aligned cells became more randomly orientated.^[^ ^231^ ^]^

##### Light Stimulation

The photoresponsive molecules, incorporated in polymer films, hydrogels, or self‐assembled monolayers (SAMs), enable to switch the chemical moieties presented on the cell culture substrate typically via either isomerization (e.g., azobenzene, nitrospiropyran) or irreversible cleavage of chemical bonds (e.g., *o*‐nitrobenzyl derivatives), resulting in the changes in ligand presentation or mechanical stiffness of the culture environment in 2D and 3D matrix (Figure 12b).^[^ ^214^ ^]^ Due to its noninvasive properties and can be precisely spatial and temporal controlled, the photo triggering method has become a popular tool for studying the dynamic crosstalk between cells and materials. For example, azobenzene has been used to fabricate switchable surfaces with reversibly displayed adhesion ligands to control cell or bacterial adhesion and detachment.^[^ ^226^, ^232^ ^]^ Azobenzene undergoes a conformational change from *trans* (*E*) to *cis* (*Z*) under UV irradiation, and recoveries by a visible light irradiation. The adhesive ligands such as RGD, sugars, or peptides are commonly coupled to the azobenzene presenting SAM coatings. The *trans*‐azobenzene displays a linear conformation that exposes the ligands for the cells to attach. When visible light is given, the *trans*‐azobenzene turns into *cis*‐azobenzene, which causes the embedment of ligands into the polymer bush structures and inhibits the physical accessibility between cells and the ligands.^[^ ^233^ ^]^

The tunable physical properties of the matrix, such as the stiffness, can be realized by integrating photoresponsive moieties. The stiffening or softening of the hydrogel can thus be regulated by photoreactions via adding or removing crosslinks from hydrogels. Compared to the introduction of chemical signals, the photocontrolled strategy allows triggering the activity at the desired time and sites. For example, a photodegradable hydrogel was prepared by incorporating the *o*‐nitrobenzyl‐based photolabile molecules into the backbone of the hyaluronic acid hydrogel. Exposure of this hydrogel to UV irradiation at any desired time or position resulted in hydrogel degradation and thus changed substrate mechanics.^[^ ^234^ ^]^

The hyaluronic acid‐based substrates capable of sequential “softening” and then “stiffening” were fabricated via incorporation of the *o*‐nitrobenzyl group and photo‐cross‐linkable methacrylate group into the backbone of HA polymers. The initial crosslinks degraded when exposed to UV light of 365 nm, resulting in a softening process (from ≈14 to 3.5 kPa) of the hydrogels. Meanwhile, the substrate softening affected the free methacrylate groups' stability. The photoinduced radical polymerization can further crosslink and stiffen the hydrogels (from ≈3.5 to 28 kPa). Cell spreading area and YAP/TAZ activities of hMSCs were decreased with the hydrogel softening, whereas they increased following the subsequent stiffening.^[^ ^215^ ^]^ It should be noticed that the commonly used photosensitive materials are most chemicals and blue light responsive, which may induce potential chemical and phototoxicity in cell culture conditions. Recently, photosensitive proteins with excellent cell compatibility and low energy green/red light responsibility emerged and attracted intensive interest in recent years. The outstanding representatives are adenosylcobalamin (AdoB_12_)‐dependent photoreceptor C‐terminal adenosylcobalamin binding domain (CarHc) proteins ^[^ ^216^ ^]^ and cyanobacterial phytochrome 1.^[^ ^235^ ^]^ The cyanobacterial phytochrome 1 exists in its monomer form under 740 nm light and switches to a dimeric state when exposed to 660 nm, leading to a reversible change in hydrogel crosslinking density. The Young's modulus of the resulting protein hydrogel can vary between 2.6 and 4.4 kPa in different photoactivating conditions.^[^ ^235^ ^]^

##### Magnetic Stimulation

The magnetic field could offer a clean, noncontact and noninvasive stimulus. Therefore, incorporating functionalized magnetic nanoparticles onto surfaces or into hydrogels has been widely considered for the design of stimuli‐responsive materials in tissue engineering. By controlling the motion of the magnetic nanoparticles via a magnetic field, magnetic force can reorganize the spatial distribution of ligands^[^ ^221^, ^222^, ^236^ ^]^ or change the tracking force between the cells and substrates.^[^ ^221^ ^]^ Bian's group developed a series of materials magnetic field‐responsive materials, and the mobility of RGD ligands that integrated is controlled by magnetic fields (Figure 12c).^[^ ^221^, ^222^, ^236^ ^]^ For instance, RGD was conjugated to the magnetic iron oxide nanoparticles linked onto a glass substrate by a long PEG chain. This long chain provided RGD ligands with a high degree of flexibility, and the flexibility can be reduced by applying magnetic attraction to these iron oxide nanoparticles. It is considered that the restricted RGD flexibility provided enhanced mechanical feedback via RGD‐integrin ligation and thus enhanced nuclear translocation of YAP, cell adhesion, and osteogenic differentiation of hMSCs. In contrast, the flexible RGD‐bearded surface limited cell spreading and osteogenesis.^[^ ^221^ ^]^

In their following study, a magnetic nanocage capable of switching “ON” or “OFF” RGD by an external magnetic field was designed to remotely regulate cell behavior in vitro and in vivo. Specifically, a magnetic cage was coupled to underlying RGD‐functionalized gold nanoparticles by using a long PEG linker. Magnetic force controlled the motions of magnetic nanocage relative to gold nanoparticles and thus allowed reversible “ON” or “OFF” of RGD for integrin binding. As expected, switching “ON” RGD significantly promoted focal adhesion formation and osteogenic differentiation of hMSCs compared to switching “OFF.”^[^ ^237^ ^]^ Macrophages (RAW 264.7 cells) were cultured on such magnetic nanocages to study the influence of physical accessibility of RGD on macrophage polarization and functions. As crucial components of the innate immune system, macrophages can be activated and polarized to different phenotypes, including the proinflammatory M1 and anti‐inflammatory M2 phenotypes. It was found that the magnetic switching “ON” RGD significantly promoted cell adhesion and M2 polarization both in vivo and in vitro.^[^ ^222^ ^]^

Very recently, Kang's group prepared RGD ligands bearing magnetic nanocoils that allowed remote mechanical stretching and shrinking of RGD ligand‐presenting nanocoils.^[^ ^238^ ^]^ These uniquely shaped ligand‐presenting nanocoils enabled the manipulations of ligand pitch in nanodimensions. Both in vitro and in vivo experiments indicated that the stretching state (magnet “ON”) fascinated significantly higher expression of FAs and adherent cell density than the shrinking state (magnet “OFF”). Higher *β*1 integrin expression was observed in the stretching state (magnet “ON”), which may fascinate the integrin ligation to the RGD bearing stretched nanocoils and enhance stem cells’ focal adhesion assembly, mechanosensing, and osteogenic differentiation. Together, these studies highlight the magnetic field as a powerful tool to manipulate mechanosensing‐mediated inflammatory or stem cell‐based tissue regeneration both in vivo and in vitro.

##### Mechanical Stretching

Cells in vivo locate in a highly dynamic environment. Due to the limitation of fabrication approaches, seldom biomaterials could offer cells, direct dynamical mechanics.^[^ ^239^ ^]^ Generally, the mechanoresponsive materials are prepared from polymer films or hydrogels and altered by applying extrinsic stress/strain stimuli (Figure 12d). The physical properties of the materials, including strain, stiffness, ligand presentation, and topography, are changed after the stimulation. PDMS is the most widely used materials in this area due to its high biocompatibility, bioinert, and tunable elasticity.^[^ ^240^ ^]^ The degrees of stretch displacement are varying in different works from 5% to 20% to match normal levels of strain observed in vivo.^[^ ^241^, ^242^, ^243^, ^244^, ^245^ ^]^

A mechanically active “lung‐on‐chip” has been developed using a PDMS‐based microfluidic device. Specifically, the PDMS channel was separated into two compartments by a thin porous PDMS membrane in the middle. Epithelial cell and endothelial cell monolayers were cultured on the upper and bottom sides of the membrane, respectively. To mimic the dynamic mechanical interactions between lung cell and their microenvironment during physiological breathing, a vacuum was applied to the side chamber to stretch/bend the PDMS film dynamically (5%–15% cyclic strain), thereby offering mechanic stress directly to the cells that grew on the membrane. The cyclic motion promoted the nanoparticle uptake and increased the transportation of nanoparticles into the microvascular channel. The nanotoxicology studies revealed that cyclic stretching force stimulus could accentuate inflammatory and toxic responses of the lung compared to that of static cases.^[^ ^246^ ^]^ This mechanics‐responsive biosystem inspires the design of mechanically active cell/organ models that provide low‐cost alternatives to animal and clinical studies.

A stretchable poly(N‐acryloyl glycinamide) (PNAGA) hydrogel^[^ ^221^ ^]^ functionalized with a quasi‐hexagonally arranged nanogold particles array coupled with RGD ligands was fabricated by our group.^[^ ^197^ ^]^ The ligand spacing can be reversibly modulated by mechanical stretching. The stretching increased inter‐ligand spacing along the stretching direction but decreased ligand spacing in the orthogonal direction. When the hydrogel was stretched to a large degree (112 nm for inter‐ligand spacing in the stretching direction, 25 nm in the orthogonal direction), the cells showed limited cell spreading and higher mobility. This result confirmed that larger ligands spacing (>73 nm) inhibits focal adhesion and integrin clustering.^[^ ^247^ ^]^ Obviously, the smaller ligand spacing (≈25 nm) in the orthogonal direction of the stretch cannot induce integrin clustering and FA mature. The limited FA formation brings unstable cell adhesion, leading to the cells’ high mobility and large migration zone. Additionally, the cell adhesion and migration could be reversibly regulated by the cyclic stretching. MSCs became more polarized and more migrated with the increase in stretch extent. Once the hydrogel was relaxed back to the initial status, the cells began to spread and sit on the surface again. It has been demonstrated that the cyclic stretch or shear stress in vitro could induce rapid reinforcement and reorientation of actin stress fibers. The unidirectional cyclic stretch also induces the rapid zyxin‐dependent mobilization of vasodilator‐stimulated phosphoprotein from focal adhesions to actin filaments, indicating that the zyxin acts as the critical mechanosensitive protein in response to the cyclic force stimulation.^[^ ^248^ ^]^

In native tissues, the function of organs such as lungs, hearts relies on the 3D stretching of their geometry. These tissue‐scale forces converge on local cellular mechanics to generate complex forms and regulate cell‐fate determinations.^[^ ^240^ ^]^ For example, the epithelial tissues often adopt a 3D architecture that forms a curved cell sheet enclosing a pressurized fluid‐filled lumen. These architectures play central roles in the development of defects, inflammatory, and cancer diseases. Recent works have demonstrated that the active superelasticity enables the epithelial tissues to undergo extreme and reversible deformations. These strains in tissues are accommodated by highly heterogeneous strains at single cell level, which are relevant to the rearrangement of cell actin cortex and filament network.^[^ ^241^ ^]^ However, how these processes are integrated and controlled across spatiotemporal scales are still unknown. One of the main reasons is that the commonly used 2D stretching system cannot mimic the 3D architecture dynamics since they require a precision control of cellular deformation, mechanical stress, and pressure. More efforts from biology and material engineering are urgently needed in this area.

##### Electrical Stimulation

The electrical potential has been widely used to alter biointerface properties.^[^ ^249^, ^250^ ^]^ The electrochemically responsive platforms could offer continuous physical stimuli by the potential‐triggered molecular conformation changes or the electrochemical oxidation‐reduction reactions. Specifically, the applied potential could induce the conformation transform or redox state of the molecules and further regulate the presentation of the ligands (also known as revere piezoelectric effect), and thereby affect the cell adhesion and migration.^[^ ^9^ ^]^ For example, the application of an oxidative potential to a conductive surface that contains hydroquinone/quinone redox couple would convert hydroquinone into a more active state benzoquinone which could covalently bond with bio‐specific ligands (e.g., RGD). These covalent bonding could be cleaved when triggered with a mild reduction potential, allowing a controllable release of bioligands.^[^ ^251^ ^]^ These electronic potential stimulative materials provide an efficient way to achieve dynamic and real‐time control over the presentation of ligands. However, these material systems deeply rely on the presence of an electrically conducting substrate and limit their application.

#### 3.2.2 Self‐Regulated Cues

The native ECM is intrinsically self‐regulated or self‐organized. It adapts biochemical and biophysical signals from cells or external stimulations and undergoes biosynthesis, degradation, and re‐modeling, impacting cell functions. These properties are summarized in **Table** **3**.

**Table 3:** Self‐regulated cues for dynamic regulation of cells

	Materials/technics	Molecular properties	Mechanisms and characteristic
Piezoelectric effect	Poly(vinylidene fluoride) Collagen^[^ ^252^, ^253^ ^]^	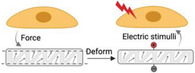	“Cell force‐electricity‐bio signaling‐cell force” loop Limited chooses of piezoelectric materials
Diffusibility	Charge/hydrophobic/supermolecule interactions^[^ ^254^, ^255^, ^256^ ^]^	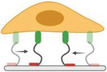	Molecular manipulation of cell force loading rate of mechanical transduction Only for 2D culture Very limited adjusting range of ligand diffusibility
Viscoelasticity	Ions/supermolecule/hydrogen bond crosslinked system^[^ ^257^, ^258^, ^259^, ^260^, ^261^, ^262^, ^263^, ^264^ ^]^	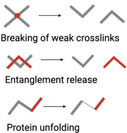	Exhibit a combination of storage of elastic energy and time‐dependent energy dissipation Affect the force loading rate of mechanical input on the integrin–receptor molecules interactions Limited material models
Degradation	Chem/biocleavable hydrogel^[^ ^265^, ^266^, ^267^ ^]^	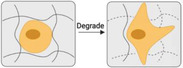	Facilitate the cellular force mediated remodeling and provide space for cell activities Very hard to control the degradation kinetics
Stress/strain stiffening	Polyisocyanopeptides hydrogel Collagen^[^ ^268^, ^269^, ^270^ ^]^	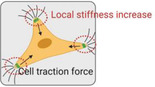	Limited material models Facilitate long‐range force transmission Narrow stiffness range (<1 kPa)

##### Piezoelectric Effect

**Figure 13:**
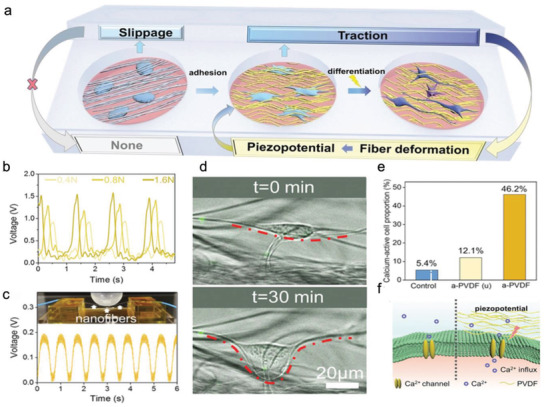
Piezoelectric materials in cell behavior regulation and sensing. a) Schematic diagram of the cell tractions induced electrical stimulus for neuron‐like differentiation. b) Cyclic mechanical stimulus triggers the voltage output of the PVDF fiber. c) The periodical force loading deforms the nano PVDF fiber bundles and generates the cyclic voltage output. d) The cell force deformed the nanofibers (fibers are labeled with FITC‐SiO_2_). e) Electrical stimulations trigger the calcium signaling activation. f) Intracellular calcium transmissions caused by the piezo potential of PVDF. Reproduced with permission.^[^ ^252^ ^]^ Copyright 2021, Wiley.

Piezoelectricity has long been proposed as one of the mechanisms underlying many biological events, ranging from ECM molecules (e.g., collagen piezoelectricity) to single‐cell activities (e.g., neuronal system activation).^[^ ^271^, ^272^ ^]^ To mimic the natural bioelectricity in cellular microenvironment, various piezoelectric biomaterials and their mediated electrostimulation have been developed to directly deliver electrostimulation to target cells and tissues. Unlike the conventional reverse piezoelectric effect‐based biomaterials (generation of force under an applied electric field) that the applied external electrical field passively manipulates cells, the direct piezoelectric materials (generation of electricity under an applied force) allow the transfer of cell tractions to an electrical stimulus that is applied on cells in situ to regulate the cell behaviors and phenotypes.^[^ ^273^ ^]^ These “cell force‐electricity‐bio signaling‐cell force” loop feedback signals enable cells to self‐regulate, providing a fancy tool to study the underlying mechanism of endogenous bioelectricity and biomechanics. Li's group developed an in situ and on‐demand electrical stimulation system based on the force‐responsive poly(vinylidene fluoride) (PVDF) nanofibers to regulate stem cell differentiation (**Figure** **13**).^[^ ^252^ ^]^ It was found that cell tractions could significantly induce the piezo potential with a range of 98 µV to 18 mV, stimulating the MSCs to differentiate into neuro‐like cells (Figure 13a–c). Notably, the electrical signals were found to activate the transmembrane calcium channel, allowing an influx of extracellular Ca^2+^ into cytoplasm (Figure 13e,f). A so‐called piezo‐phototronic light nanoantenna array based on InGaN/GaN was established in the following study to achieve real‐time mapping of living cell traction force.^[^ ^253^ ^]^ The photoemission properties of InGaN/GaN nanopillar are susceptible to their inner piezo‐potential dynamics. The cell traction force can be directly captured and visualized at a spatial resolution of 800 nm and a temporal resolution of 333 ms. These results highlight the piezoelectric biomaterials in cell behavior modulating.

##### Diffusibility

The dynamic cell–matrix interactions cause ligand diffusion at the adhesive interfaces. Capturing the dynamic spatial presentation of biochemical molecules enables us to explore the receptor‐mediated intracellular signaling in responding to the varying cellular microenvironment. Supramolecular‐based surfaces (e.g., lipid bilayer surfaces and polymer surfaces) have been used to establish artificial self‐dynamic systems. Viscosity defines the range of motion of the molecules on a substrate. Supramolecular surfaces composed of different molecules exert different viscosity and could offer additional molecular flexibility, binding affinity, ligand density, and ligand mobility for cells, and therefore can influence cellular behaviors such as cell adhesion,^[^ ^11^ ^]^ spreading,^[^ ^274^ ^]^ focal adhesion formation^[^ ^275^ ^]^ and differentiation.^[^ ^274^, ^276^ ^]^

Supported lipid bilayers (SLBs) are supramolecular architectures formed by vesicle fusion on a hydrophilic surface. They are designed to mimic the cell membrane system, providing a well‐characterized and easily manipulated system to study cell behavior in response to mobile and viscous matrix properties.^[^ ^277^ ^]^ By controlling their base lipid content and functional groups, SLBs show multiple physicochemical characteristics which are essential for their applications in biofield. For example, the zwitterionic group on the head group of the base lipids provides a protein and cell‐resistant surface (i.e., nonfouling). This ability enables the cells to specifically interact with the bio‐ligands incorporated onto the SLBs.

**Figure 14:**
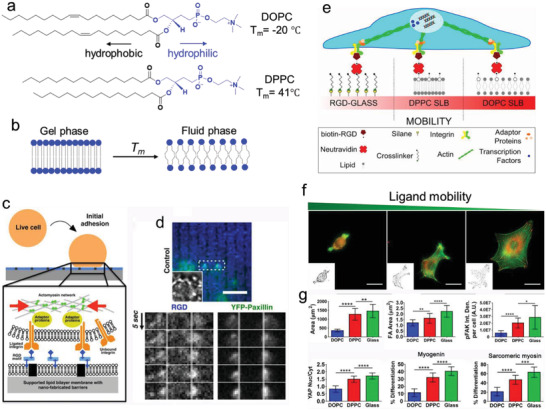
Ligand mobility regulates cell adhesion and mechanotransduction. a) The chemical structure of DOPC and DPPC. b) Working principle of phase transition of lipid bilayer responding to the critical temperature *T*_m_. When the temperature exceeds the threshold temperature, the carbon chins melt and the order the lipid packing will significantly decrease, resulting in an increased fluidity. c) The scheme of nano‐Patterned RGD functionalized fluidic SLBs. d) The time‐lapse of the RGD and YFP‐paxillin localization at the contractile clusters during early stage of cell adhesion. The images show that the RGD and paxillin aggregate against the nanoscale barriers (Scale bar indicates 5 µm). Reproduced with permission.^[^ ^256^ ^]^ Copyright 2011, National Academy of Sciences. e) DOPC and DPPC lipid bilayers with distinct diffusibility for cell adhesion study. f) The fluorescent images of cell spreading on the surfaces with different ligand mobility (green: actin; red: vinculin, scale bar: 25 µm). g) The quantitative data of C2C12 cell spreading area, focal adhesion, pFAK, YAP nuclear localization, and cell differentiation. Reproduced with permission.^[^ ^255^ ^]^ Copyright 2018, National Academy of Sciences.

The phase behavior of SLBs highly correlates with the characteristic melting temperatures of the lipids, which is determined by their alkyl tail structures. SLBs consist of two commonly used lipids, one with a low melting temperature (e.g., 1,2‐dieoleoyl‐sn‐glycero‐3‐phosphocholine, DOPC, *T*_m_ = ‐20 °C) presents high ligand mobility. In contrast, the other with high melting temperature (e.g., 1,2‐dipalmitoyl‐sn‐glycero‐3‐phosphocholine, DPPC, *T*_m_ = 41°C) offers fewer mobile ligands at physiological temperatures (**Figure** **14**a,b). The viscosity of SLBs can be modulated, and it can be easily modified with different functional groups, making SLBs a popular material in the study of ligand mobility and clustering.^[^ ^254^, ^278^, ^279^ ^]^ Koçer and Jonkheijm fabricated SLBs with different RGD ligands diffusibility.^[^ ^276^ ^]^ The highly diffusible surfaces enhanced the cell adhesion, spreading, proliferation, and osteogenic differentiation of MSCs. However, by using similar dynamic SLB systems, Bennett et al. found that the viscosity of the bilayer drives the mobility of the ligands presented on the surface. Increasing ligand mobility led to a decreased surface viscosity and a monophasic change of cell morphology to a smaller and round shape, resulting in a decreased cell tension, YAP activity, and myogenic differentiation (Figure 14e–g).^[^ ^255^ ^]^ Direct comparisons may not be drawn due to the distinct differences in the protocols and cells used in these studies. Not only the ligand diffusion kinetics, but also the traction force, ligand intermolecular distance, and other surface chemistry properties (e.g., charge), as well as the cell force‐sensing range, cause the debatable results in different experimental conditions.^[^ ^248^, ^252^ ^]^

SLBs have served as an excellent model to investigate the integrin‐based cell signaling. Fluidic DOPC SLBs were functionalized with RGD via biotin–neutravidin interactions and physically separated by nano‐sized barriers (Figure 14c). The integrin–RGD interaction induced the formation of sub‐micron sized integrin clusters. At the same time, the integrin‐*β*3 and RGD ligands were gradually colocalizing during the first 200 s of adhesion. Interestingly, multiple cytoplasmic adaptor proteins, including paxillin, talin, and FAK, were recruited to the cluster site in a force‐independent manner since the recruitment was not inhibited by actomyosin inhibitors. Subsequently, the formation of the integrin‐RGD cluster caused remodeling of the actin network and stimulated local actin polymerization where actin filaments grew from the early clusters (nascent adhesions). These actin‐enriched nascent adhesions were then observed to move laterally towards each other and pile up against the nano barriers (Figure 14d). Compared to the continuous SLBs, the nanobarriers separated SLBs activated cell spreading more efficiently. A higher density of these nano‐barriers can significantly promote cell spreading, possibly due to the higher density adhesion sites next to the nanobarriers.^[^ ^256^ ^]^

Amphiphilic block copolymers have been applied to prepare diffusible surfaces to realize the dynamic display of ligands and offer support to cell adhesion/spreading. A vital characteristic of the amphiphilic block copolymers is that they can organize into various structures in response to the different solvent conditions. Compared with the aggregation behavior of small surfactants and lipid molecules, the amphiphilic block copolymers resemble to form stable structures due to their larger molecular weight.^[^ ^280^ ^]^ In addition, their size and block copolymer chains are controllable, allowing the independent tuning of their mobility and mechanical properties.^[^ ^275^, ^281^ ^]^ For instance, the lateral mobility could be changed via adjusting the proportion of a “lubricating” homopolymer.^[^ ^281^ ^]^ Similarly, the ligand density could be easily tuned by the fraction of the RGD functionalized polymers. Kourouklis et al. created self‐assembled films with tunable lateral mobility using amphiphilic block copolymers 1,2‐polybutadiene‐*b*‐poly(ethylene oxide) (PB‐*b*‐PEO). RGD peptides were added to the termination of the block copolymer to allow cells to adhere, and a trace hydrophobic homopolymer (poly(isobutylene) PIB) was added to control the lateral film mobility. A dynamic bio‐surface with varied mobility was thus developed through the controlling PIB fraction. At low mobility, the cellular contractions were efficiently sustained, facilitating the formation and maturation of FAs. At high mobility, ligand‐integrin interaction occurred faster and more efficiently, resulting in a higher density of FAs but limited FAs size. The integrins inside FAs diminished at the substrate with high mobility, but the integrins outside FAs collectively supported cell adhesion during cell spreading on diffusible ligands.^[^ ^275^ ^]^ In the following study, the role of different integrins on cell adhesion over mobile films was investigated. Specifically, *α*5*β*1 and *α*v*β*3 integrins induced ligand diffusion‐dependent effects on cell spreading and polarization. The cells prefer to employ *α*v*β*3 integrins to promote cell spreading and polarization on the films with low ligand mobility, while *α*5*β*1 integrins favor the films with high ligand mobility. The accumulation of *α*v*β*3 integrins in FAs enabled the increase of FAs. The spreading, particularly associated with the ligation and clustering of *α*5*β*1 integrins, led to the small size of FAs.^[^ ^282^ ^]^

**Figure 15:**
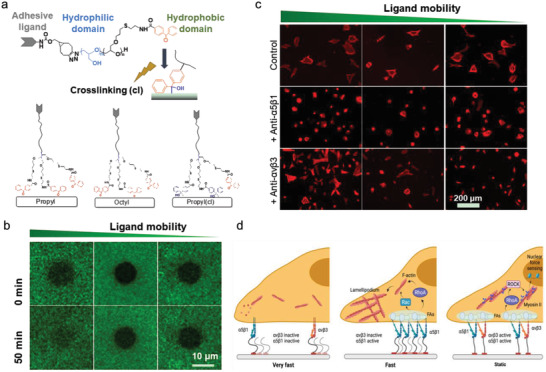
Ligand diffusion regulates cell adhesion in an actomyosin force‐independent manner. a) Scheme of the working principle of diffusible monolayer polymer coatings. b) Representative images of FRAP measurement of fluorescence‐labeled ligands with different mobility. c) Representative images of cells after treatment with *α*5*β*1 or *α*v*β*3 integrin antibodies for 4 h (Red: F‐actin). d) The distinct signaling pathways for cells on ligand diffusible surfaces. Specifically, the fast ligand diffusion enables cell to recruit *α*5*β*1 integrin, activating *α*5*β*1 integrin and initiating Rac and RhoA signaling to promote cell adhesion but not osteogenesis. Whereas the slow/constant ligand diffusion surface provide larger cell tractions. It can activate myosin II and the RhoA/ROCK signaling pathway, resulting in an enhanced cell spreading and osteogenesis. Reproduced with permission.^[^ ^283^ ^]^ Copyright 2020, Wiley‐VCH.

To explore the integrin‐dependent cellular mechanosensing signaling pathway involved in dynamic integrin‐ECM interactions, we recently developed a set of model surfaces with controllable ligand diffusion via self‐assembling polyglycerol‐based amphiphilic block copolymers on the substrates with distinct hydrophobicity.^[^ ^254^ ^]^ The polymer diffusibility can be tuned by adjusting the hydrophobic interactions between the hydrophobic domain of the ligands and the substrates (**Figure** **15**a). The diffusivity of the ligands was quantified by fluorescence recovery after photobleaching (FRAP) (Figure 15b). The surface morphology, wettability, and ligand density were kept consistent. The surface background was bioinert to exclude any nonspecific interaction. Only the ligand‐coupling strength was adjusted to regulate ligand diffusion. Interestingly, the cellular sensing of ligands mobility is integrin types dependent (Figure 15c). The cells on the highly mobile surface could recruit the ligands via *α*5*β*1 integrins that further activated RhoA and Rac pathways, resulting in an enhanced lamellipodia formation and cell spreading but limited osteogenesis of MSCs. Meanwhile, the restrictive ligands induced the crosstalk between *α*v*β*3 and *α*5*β*1 integrins. They initiated the RhoA/ROCK pathway to activate myosin II, leading to stress fiber‐based cell adhesion and the osteogenic differentiation of MSCs (Figure 15d). These findings were consistent with the previous studies that cells can sense substrate mobility by selectively activating *α*5*β*1 and *α*v*β*3 integrins. In the follow‐up study, we established a bio‐mimicked self‐strengthening monolayer polymer coating based on photosensitive spiropyran to mediate cell mechanosensing and mechanotransduction. The ligand diffusibility decreased over time with the spontaneous merocyanineto‐spiropyran (MC‐SP) thermal isomerization. The Rac signaling and RhoA/ROCK signaling were subsequently activated via progressive activation of *α*5*β*1 and *α*v*β*3, respectively, from MC state to SP state, accompanied by the enhancement of stem cell mechanotransduction and osteogenesis.^[^ ^283^ ^]^

##### Viscoelasticity—Stress Relaxation

**Figure 16:**
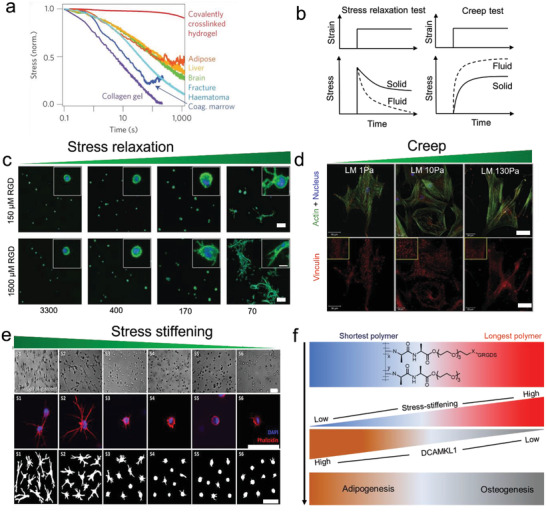
Influence of nonlinear mechanical properties on cellular mechanoresponse. a) Overview of stress‐relax properties of different tissues and hydrogels. Reproduced with permission.^[^ ^284^ ^]^ Copyright 2012, Nature Publishing Group. b) Viscoelastic materials exhibit stress relaxation in response to a constant deformation and increased strain, or creep, in response to constant stress. Reproduced with permission.^[^ ^287^ ^]^ Copyright 2020, Nature Publishing Group. c) The faster stress relax properties of hydrogels enhance the cell spreading and proliferation.^[^ ^284^ ^]^ Scale bar indicates 20 µm. Reproduced with permission.^[^ ^284^ ^]^ Copyright 2012, Nature Publishing Group. d) Immunohistochemical stains of actin filaments (green color) and focal adhesions (vinculin, red color) for cells on the substrate with varied creep properties. Scale bar indicates 50 µm. Reproduced with permission.^[^ ^263^ ^]^ Copyright 2011, Elsevier. e) The bright field and fluorescence images of hMSCs spreading in the PIC matrix with various stress‐stiffening properties. Red: F‐actin stained with phalloidin, blue: nucleus stained with DAPI. Reproduced with permission.^[^ ^302^ ^]^ Copyright 2019, American Chemical Society. Scale bar indicates 70 µm. f) Stress stiffening regulates the adipogenesis and osteogenesis of stem cell by modulating the expression of DCAMKL1.^[^ ^268^ ^]^ Reproduced with permission.^[^ ^268^ ^]^ Copyright 2016, Nature Publishing Group.

The natural ECMs are not ideally elastic. They are viscoelastic and exhibit stress relaxation in response to an applied strain: the initial stress resisting an applied strain decreases over time due to reorganization processes that relax the stresses in the matrix (**Figure** **16**d).^[^ ^15^, ^284^ ^]^ For example, various organs and tissues, such as liver, brain, adipose, bone marrow, and initial fracture hematomas, are viscoelastic and exhibit partial stress relaxation (Figure 16a). Cells exert forces on the surrounding environment. In an elastic matrix, most energy exerted by the cells is stored within the static elastic networks, and the microenvironment keeps constant. In contrast, in a viscoelastic matrix, the stress relaxation of the substrate would release pent‐up energy to eliminate the cells’ force, resulting in matrix deformation and allowing cells to undergo spreading, polarization, and migration.^[^ ^257^ ^]^ The stress relaxation rate is highly correlated with the matrix deformation degree. The faster it relaxes, the larger remodeling it will have. These self‐regulated mechanical cues have a significant effect on cell behaviors.^[^ ^258^ ^]^ However, how the stress relaxation regulates cellular behaviors is still unknown.^[^ ^15^ ^]^

The mechanisms underlying the dissipative properties of ECM or tissues have been investigated. The native ECM is consisting of numerous fibrous protein networks. The typical example is the collagen fibers which integrated with highly hydrated, flexible polysaccharides, playing a vital role in regulating tissue mechanics and energy dissipation. Most of the network bindings are noncovalent, of which dissociation rates are rapid enough to allow stresses to relax or allow materials to creep. The resulting dissociated or reformed weak bonds can stabilize the deformed state of materials. On the other hand, the energy dissipated in the proteins results in protein unfolding, further enabling the energy to dissipate under external stress.^[^ ^259^ ^]^

The biomaterials with stress relaxation properties have been designed. The commonly used strategy is to integrate stable and bioinert polymers with weaker interactions or entanglement interactions into more stable covalent crosslinking systems. Once the entangled polymer chains are released, the energy will dissipate, and the whole matrix will relax. Other approaches based on weak chemical crosslinking strategies, such as ionic crosslinking, boronate bonds, or thioester exchange, have also been utilized to form the hydrogel systems with various stress relaxation properties.^[^ ^259^, ^260^, ^261^ ^]^ Additionally, as biomaterials such as hydrogels exhibit a high‐water content, the movement of fluid within the matrix may lead to the dissipation of energy.^[^ ^261^ ^]^ Recently, Chaudhuri et al. fabricated an alginate hydrogel system with controllable stress relaxation property for natural viscoelasticity mimicking.^[^ ^284^ ^]^ The stress relaxation property of the alginate hydrogels can be altered by: molecular weight of alginate, different crosslinking densities of Ca^2+^, and the length of PEG spacer. These hydrogels remained nearly the same initial elastic modulus. It was found that long timescales of stress relaxation (3300 s) could efficiently suppress both cell spreading and proliferation of 3T3 cells. However, faster‐relaxed hydrogels (70 s) significantly enhanced cell spreading and proliferation (Figure 16c). The fast‐relaxing matrices can be deformed by cells, allowing increased clustering of embedded integrin motifs, leading to increased integrin adhesion, Rho activation, myosin‐actin contractility, and YAP nuclear‐cytoplasmic translocation, and thus, promoted spreading and osteogenesis of MSCs (Figure 16b).^[^ ^285^ ^]^ Compared to the cells on the purely elastic substrate with the same elasticity, the cells on viscoelastic substrates generate more work since the energy applied to the matrix is dissipated, which may enable cell spreading.^[^ ^285^, ^286^ ^]^ Alternatively, the stress relaxation has been proved to enhance the lifetime of integrin–ECM interactions potentially. Thus, the viscoelastic materials decrease the force applied to individual bonds and increase the lifetime and stability of slip bonds and eventually enhance the cellular mechanotransduction.^[^ ^72^, ^287^ ^]^

The matrix with proper stress relaxation property has been applied in tissue engineering. In MSC‐based therapy for calvarial defects, new bone grew significantly faster in the rats treated with MSCs encapsulated in fast‐relaxing hydrogels than slow‐relaxing gels.^[^ ^288^ ^]^ The rapid stress relaxation of the hydrogels could enhance the cartilage matrix production.^[^ ^289^ ^]^ Overall, these studies highlighted the matrix stress relaxation in the development of new biomaterials and their applications in tissue engineering.

##### Viscoelasticity—Creep

As discussed above, viscoelastic materials respond as both elastic solid and viscous fluid. This behavior yields time‐dependent mechanical properties, including stress relaxation in response to deformation, and creep in response to applied mechanical stress.^[^ ^290^, ^291^ ^]^ The time‐dependent dissipation of energy after the initial matrix deformation (creep) should not be ignored in cell mechanoresponse. At a constant elastic modulus, the higher the matrix's viscosity, the more obvious time‐dependent deformation incurred under a mechanical stimulus.^[^ ^292^ ^]^ In contrast to a purely elastic substrate, the viscoelastic substrate will creep when a pulling force is applied, and the cells may feel a time‐dependent reduction in the resistive force. Thereby creep impacts not only the maturation of FAs but also the downstream signaling processes.^[^ ^46^ ^]^ PAAm gels are often used to study the cellular response to the viscous property. The viscous degree of PAAm materials can be regulated through adjusting the proportion of linear polymers with high molecular weight. The polymers would be sterically entrapped in the hydrogels,^[^ ^262^ ^]^ or simply adjusting the ratio of acrylamide monomer and bis‐acrylamide.^[^ ^263^ ^]^ Cameron et al. found that the increase of gel loss modulus will lead to an increase of creep, promoting the cell spreading, proliferation, and differentiation (Figure 16e). The cells would lose passive tension when they apply a force to high viscous substrates that creep to dissipate the energy. To maintain “tensional homeostasis,” the cells would require increased active tension (actin‐myosin contractility). However, this increase in active tension alone would be insufficient to maintain balance. Whereas increased isotonic tension, generated by cell spreading or locomotion, would effectively restore the forces to remain their mechanical homeostasis.^[^ ^263^ ^]^ The follow‐up study indicated that hMSCs on the substrate with higher creep exhibited enhanced GTPase Rac 1 activation, cell motility, and lamellipodial protrusion rate. The dynamic creep properties will continually create imbalance at the cell leading edge of cell where the traction force was applied. However, the depleted trace force caused by substrate creep can be compensated by an enhanced protrusion rate, which promoted lamellipodia formation, mechanosensing and further facilitated cell migration.^[^ ^264^ ^]^

##### Degradation

The ECM components, such as collagen and fibrin, are enzymatically degradable. This process encourages ECM to release matrix‐tethered biomolecules to regulate cell functions. Meanwhile, the degradation causes scaffold remodeling that allows the cell to migrate. The most significant enzymes involved in ECM remodeling in the natural scaffold are metalloproteinases, especially matrix metalloproteinase (MMP) family and a disintegrin and metalloproteinase with thrombospondin motifs (ADAMTS) family.^[^ ^293^, ^294^, ^295^ ^]^ Compared with naturally derived biomaterials, the synthetic polymers could offer better batch‐to‐batch consistency and quality control.^[^ ^296^ ^]^ Various biochemical reactions have been utilized in the preparation of degradable biomaterials, such as hydrolysis (esters, anhydrides, and thioesters), enzyme‐sensitive degradation (MMP degradable crosslinkers or peptides), and stimuli‐sensitive degradation (photodegradable systems).^[^ ^297^ ^]^ Cells require growing space and proper external mechanical stimulation to induce and regulate cell functions. Therefore matrix degradation is essential for cell activities, especially in 3D microenvironments.^[^ ^116^ ^]^ Nondegradable or space‐restrictive stiff 3D hydrogels suppress cell spreading, growth, and osteogenic differentiation due to the lack of enough space induced by the dense crosslinking networks. While the adaptable degradable hydrogels efficiently enhance the cell spreading and functions in the 3D environment. For example, MSCs displayed chondrocyte morphology and expressed a high level of chondrogenic markers in degradable gels fabricated by methacrylated hyaluronic acid hydrogel crosslinked with MMP degradable crosslinkers. In comparison, MSCs in nonsensitive hydrogels showed limited cell spreading with round morphology.^[^ ^298^ ^]^ Khetan and Burdick reported similar results: cells could not spread in highly crosslinked hydrogels with nondegradable networks.^[^ ^299^ ^]^

The ideal degradable scaffold materials for tissue engineering are those whose degradation is elaborated by seeded cells. Cell‐compatible hydrogels have been designed to degrade via enzymatic hydrolysis induced by cell‐secreted enzymes. Burdick et al. developed a 3D covalently sequential cross‐linked HA hydrogel by primary addition (degradable MMP peptides) and secondary radical polymerization (nondegradable) strategy. Compared to the restricted hydrogels with the same elastic modulus, degradable hydrogel permitted a higher degree of cell extension, traction force, and osteogenesis. The surrounding matrix was thus deformed by cells to a greater extent, which further enhanced the cell adhesion, cytoskeletal organization, and mechanosensing.^[^ ^265^ ^]^

The hydrogel degradation rate is quite critical in regulating cell functions, even exceeding the effect of hydrogel stiffness. Recently, Ding et al. prepared the degradable 2D PEG hydrogel and found fast degradation promotes the osteogenesis of hMSCs on soft substrate.^[^ ^266^ ^]^ Heilshorn et al. prepared 3D degradable elastin‐like protein hydrogels functionalized with RGD peptides and found that stemness maintenance was not correlated with initial hydrogel stiffness or cytoskeletal tension generation, but strongly sensitive to the degradability of the hydrogels. Degradable hydrogels enable cell to remold the matrix mechanics, allowing neural progenitor cell self‐renewal and potency.^[^ ^267^ ^]^

Together, these studies illustrated the importance of degradability in regulating cellular functions in 3D culture systems. However, it remains challenging to control degradation kinetics that can match the cellular timescales and the formation of degradation byproducts.^[^ ^15^ ^]^

##### Strain/Stress‐Stiffening

The fibrous biopolymers, such as fibrin, F‐actin, microtubules, collagen, or elastin that form networks within the cytoskeleton or ECM, show nonlinear elastic behavior manifested in tension‐strain‐stiffening (Figure 16f).^[^ ^300^, ^301^ ^]^ These biopolymer networks become several times stiffer when a small strain is applied.^[^ ^302^ ^]^ This matrix property facilitates the long‐range force transmission, which enable cells to sense and respond to mechanical signals from a distance.^[^ ^303^ ^]^ On nonlinear strain/stress‐stiffening fibrin gels, the forces can be transmitted between fibroblasts or MSCs as far as ≈500 µm (≈30 cell diameters).^[^ ^304^ ^]^ In contrast, the cells adhered on hydrogels with linear elasticity can sense and respond to the force loading generated by neighboring cells within ≈25 µm.^[^ ^305^ ^]^ Furthermore, both fibroblasts and hMSCs can stiffen the fibrin gels by applying myosin‐based contractions that drive the gels into a stiff nonlinear elastic regime. The cells will, in tune, increase their internal mechanics to adapt the underlying matrix and thus result in elongated morphology, enhanced cell spreading, and migration.^[^ ^306^ ^]^ Therefore, a matrix with strain/stress‐stiffening property has been developed to guide cell behaviors. The unique representative of stress stiffening material is polyisocyanopeptide (PIC) hydrogels which consist of a *β*‐helical architecture. These structures are stabilized by a peptidic hydrogen bond network along the polymer backbone. The stress stiffening property of PIC hydrogels could be adjusted by molecular structure, PIC concentration, and external conditions, such as temperature and salt concentration.^[^ ^269^ ^]^ For instance, PIC hydrogels with high concentration displayed suppressed stiffening response, while softer PIC gels were more stress‐responsive as they stiffened at lower stresses.^[^ ^302^ ^]^ This was further confirmed by in vitro experiments that cells encapsulated in low gel concentrations showed significantly better spreading than those cultured in PIC with higher concentrations (Figure 16h). It should be noticed that the initial stress‐sensitive PIC hydrogels are very soft (<1 kPa). They can be maximally stiffen up to kilopascals that are mostly accessible in the native cell environment.^[^ ^302^ ^]^ Das et al. fabricated soft thermo‐responsive PIC hydrogels at a low concentration (0.05% wt). The nonlinear stress mechanics were precisely controlled by the macromolecular length and density of the bifunctional group.^[^ ^268^ ^]^ The increased polymer length caused enhancement of critical stress, resulting in increased osteogenic differentiation of encapsulated MSCs. A microtubule‐associated protein, DCAMKL1, was suggested to contribute to this strain/stress‐stiffening‐mediated mechanotransduction pathway (Figure 16g).^[^ ^268^ ^]^

## 4 Conclusion and Outlook

In this review, we highlight the novel materials used to mimic the mechanic cues of native ECM. In particular, the mechanic signals from ECM are classified into static and dynamic cues. The generation and transmission of these mechanic cues into the cells and cell responses have been systematically discussed. We emphasize the active force‐biosignaling feedback loops in the dynamic cell–matrix interactions. Having reviewed the latest progress in this field, we conclude the challenges and potential directions from the biomaterial design, and corresponding cell mechanosensing mechanisms. We anticipate that these may facilitate the mechanobiology study toward various biomedical applications.

Most of the vital biological processes occur in proper time and spatial scales. For instance, stem cell differentiation takes place on the order of tens of days, while cells migrate in several micrometers per minute. It thereby requires the dynamic biomaterials to match various time and spatial scales where the biological processes occur. Meanwhile, the mechanics of natural cellular microenvironments gradually evolve during development or disease progression. It certainly influences the cellular response and functions over time. Therefore, biomaterials simply offering invariable biomechanical features are far enough to exact regulate cell behaviors. The advancing biomaterials have called for the matrix with features of self‐adaptability, which allows the system to maximize the synergistic effect of matrix and cells.

Cells sense mechanical information as a systematic process involving diverse signaling factors at various cellular compartments in different periods. Less is known about the crosstalk among numerous physical or biochemical cues at the molecular level. To address these issues, a few studies employed combined strategies to design the substrate with multiple parameters. These provide more insights into the understanding of the complex cellular microenvironments. However, these studies have investigated limited contexts such as “elasticity and topography,” “elasticity and ligand presentation,”,“elasticity and growth factors,” and etc., which cannot cover the fundamental aspects of the cellular microenvironment. New stimulative materials and technologies may assist in building the native microenvironment and manipulating or capturing the cell dynamics. On the other hand, the current methods cannot capture the intricate feedback loops between the resident cells and their surrounding ECM. More studies are needed to develop efficient stimulative technologies or materials for practical applications.

The current knowledge about cell mechanotransduction in micro/nanospatial scale microenvironment is limited. Most of the interactions between the cells and the matrix are expected to dynamically alter the local matrix architecture, viscoelasticity, or ligand distribution on the tiniest scale. Hence, more advanced sensors and approaches that allow cell–materials interactions to be deciphered with a higher spatiotemporal resolution are desired. For example, piconewton level molecular tension sensor, super‐resolution imaging system, etc. These technics allow a direct readout of the force dynamics at the cell–matrix biointerface in molecular scale. Future studies may focus on the new biosensing technologies to achieve in situ, real‐time, and even long terms of cell tracking in 2D and 3D dimensions.

Additionally, our understanding of the mechanotransduction signaling pathways remains to be improved. Even significant progress has been achieved in connecting the physical cues and cell signaling. Studies mainly focused on integrin‐based mechanosensitive receptors, myosin‐based contractility, and YAP/TAZ nuclear localization. Mechanosensing and mechanotransduction processes are more complicated. Questions such as how cells sense and respond to the mechanical forces exerted from their adjacent cells; how the other bioactive macromolecules (e.g., lncRNA, miRNA) participate in the mechanotransduction process; how various bioactive macromolecules cooperate in the mechanotransduction process; in different cell types, mechincal forces lead to different cell function through distinct signaling pathways, how mechincal forces affect stem cell differentiation, immune cell migration and activation remain challenging.

The design of traditional biomaterials has historically operated without considering the dynamic feedback loops between cells and the matrixes. It is mainly due to the poor knowledge about the detailed molecular dynamics (e.g., adhesion proteins unfolding and interactions, turnover, and lifetime dynamics) and the sophisticated force interactions at the cell‐matrix interface. Additionally, the lacking of the proper intelligent materials or characterization technologies to decouple the complex interactions between various physical cues with high spatiotemporal resolution cannot be denied. Therefore, we anticipate that more advanced biomaterials and technologies will be used to reveal the real‐time “resonance” between cellular signaling and matrix mechanics. We believe this will significantly promote the development of biomaterials in tissue engineering and regenerative medicine.

## Conflict of Interest

The authors declare no conflict of interest.
